# Dissecting the role of the gut microbiome and fecal microbiota transplantation in radio- and immunotherapy treatment of colorectal cancer

**DOI:** 10.3389/fcimb.2023.1298264

**Published:** 2023-11-16

**Authors:** Lena Van Dingenen, Charlotte Segers, Shari Wouters, Mohamed Mysara, Natalie Leys, Samir Kumar-Singh, Surbhi Malhotra-Kumar, Rob Van Houdt

**Affiliations:** ^1^ Nuclear Medical Applications, Belgian Nuclear Research Centre, SCK CEN, Mol, Belgium; ^2^ Laboratory of Medical Microbiology, Vaccine and Infectious Disease Institute, Faculty of Medicine, University of Antwerp, Antwerp, Belgium; ^3^ Molecular Pathology Group, Laboratory of Cell Biology and Histology, Faculty of Medicine, University of Antwerp, Antwerp, Belgium; ^4^ Bioinformatics Group, Center for Informatics Science, School of Information Technology and Computer Science, Nile University, Giza, Egypt

**Keywords:** colorectal cancer, radiotherapy, immunotherapy, fecal microbiota transplantation, microbiota

## Abstract

Colorectal cancer (CRC) is one of the most commonly diagnosed cancers and poses a major burden on the human health worldwide. At the moment, treatment of CRC consists of surgery in combination with (neo)adjuvant chemotherapy and/or radiotherapy. More recently, immune checkpoint blockers (ICBs) have also been approved for CRC treatment. In addition, recent studies have shown that radiotherapy and ICBs act synergistically, with radiotherapy stimulating the immune system that is activated by ICBs. However, both treatments are also associated with severe toxicity and efficacy issues, which can lead to temporary or permanent discontinuation of these treatment programs. There's growing evidence pointing to the gut microbiome playing a role in these issues. Some microorganisms seem to contribute to radiotherapy-associated toxicity and hinder ICB efficacy, while others seem to reduce radiotherapy-associated toxicity or enhance ICB efficacy. Consequently, fecal microbiota transplantation (FMT) has been applied to reduce radio- and immunotherapy-related toxicity and enhance their efficacies. Here, we have reviewed the currently available preclinical and clinical data in CRC treatment, with a focus on how the gut microbiome influences radio- and immunotherapy toxicity and efficacy and if these treatments could benefit from FMT.

## 1 Introduction

Colorectal cancer (CRC) is the third most commonly diagnosed cancer and the second cause of cancer-related deaths worldwide (Sung et al., 2021). The highest incidence of CRC can be found in Europe, North America and Oceania, which is likely due to lifestyle factors, i.e., less physical activity, consumption of high-calorie-dense food, and smoking (Center et al., 2009). Indeed, the latter have been identified as CRC risk factors, next to age, gender, family history, colitis, alcohol consumption, high consumption of red and processed meat, obesity and diabetes (Taylor et al., 2010; Chan et al., 2011; Fedirko et al., 2011; Jiang et al., 2011; Jess et al., 2012; Ma et al., 2013). On the other hand, there are also elements that are associated with a lower chance to develop CRC, such as physical activity, a healthy diet, removal of precancerous lesions, hormone replacement therapy, and aspirin (Brenner et al., 2011; Bosetti et al., 2012; Boyle et al., 2012; Lin et al., 2012; Veettil et al., 2021). However, the reported lower incidence of CRC in Asia and Africa might also be attributed to poor access to healthcare and screening tools (Pourhoseingholi, 2012; Awedew et al., 2022).

Treatment of CRC is based on tumor- (e.g., tumor size/progression, presence and localization of metastases) and patient-related factors (e.g., prognosis, general health, age) (Mármol et al., 2017). Generally, the standard course of treatment for CRC is surgery (Kuipers et al., 2015). Neoadjuvant (before surgery) and adjuvant (after surgery) therapies mostly involve chemotherapy, radiotherapy or a combination of both (Brenner et al., 2014; Kuipers et al., 2015). However, because treatment is adapted to the patient during the course of treatment, it is difficult to define the percentage of patients receiving a certain treatment course.

Radiotherapy is an important treatment option for CRC, but is also associated with acute and/or chronic toxicity (Häfner and Debus, 2016; Segers et al., 2019). Acute symptoms of pelvic radiotoxicity are diarrhea, nausea, fatigue and abdominal pain (Hauer-Jensen et al., 2014). Chronic pelvic radiotoxicity is associated with changes in intestinal transit, malabsorption, impaired gut motility, fistula formation, intestinal obstruction and perforation (Hauer-Jensen et al., 2014; Segers et al., 2019). These side effects can be so severe that treatments need to be stopped temporarily or even permanently. The risk to develop radiation-related side effects is partly therapy- (e.g., radiation dose, fraction, site, and concomitant treatments) and partly patient-related (e.g., sex, age, genetic susceptibility, and smoking) (Bentzen and Overgaard, 1994; Andreassen and Alsner, 2009). In addition, radiotherapy can disrupt the gut microbiome, which in turn can influence radiotherapy efficacy by hindering the ability to repair radiation-induced intestinal damage (Liu et al., 2021).

Other emerging therapeutics are also being applied on CRC. As cancer cells are able to evade the immune system, there is a scientific ground to target the tumor by blocking these immune evasion mechanisms or activating the immune system (Kim and Cho, 2022). Furthermore, the presence of T-cells in the CRC tumor microenvironment is associated with better prognosis, indicating that targeting T-cells might be useful for this cancer type (Galon et al., 2006; Galon et al., 2007). Therefore, the use of immunotherapy has been explored for the treatment of CRC (Ganesh et al., 2019). More specifically, immune checkpoint blockers (ICBs) that enhance T-cell activation by blocking the co-inhibitory T-cell receptor programmed cell death 1 (PD-1) or its ligand (PD-L1) or cytotoxic T lymphocyte antigen 4 (CTLA-4) have shown promise. Based on the KEYNOTE-177 and CheckMate 142 clinical trials, the anti-PD-1 molecules pembrolizumab and nivolumab, and the combination of nivolumab and the anti-CTLA-4 molecule ipilimumab have been approved by the Food and Drug Administration (FDA) for CRC treatment (Overman et al., 2017; Diaz et al., 2022; Lenz et al., 2022). An important predictive marker for ICB efficacy is microsatellite instability (MSI). Microsatellites are short tandem repeat DNA sequences that are more susceptible to replication defects and are normally corrected by mismatch repair (MMR) (De’ Angelis et al., 2018). An incorrect repair by the MMR system results in the MSI phenotype with the generation of frameshift mutations and synthesis of neoantigens, making cancer cells more easily detectable by the immune system (Maby et al., 2016). Based on their frequency, three different microsatellite statuses have been characterized: high MSI (MSI-H), low MSI (MSI-L) and microsatellite stability (MSS) (Bonneville et al., 2020). Patients who are MSI-H can also be identified as MMR deficient (dMMR), whereas patients who are MSI-L or MSS can be identified as MMR proficient (pMMR) (Sinicrope and Yang, 2011). ICBs are only applicable for MSI-H CRC patients, since MSI-L and MSS CRC patients are unresponsive to ICBs due to the lack of tumor mutational burden, little tumor-infiltrating lymphocytes (TILs), low PD-L1 expression on tumor cells and low IFN-γ expression (Galon et al., 2006; Le et al., 2017; Ganesh et al., 2019). However, only 15% of all CRC patients are MSI-H, suggesting that only a small subset of patients could benefit from immunotherapy (Boland et al., 1998). Furthermore, increasing evidence suggests that the gut microbiome is another important factor influencing anti-tumor responses and ICB efficacy in CRC (Hou et al., 2022).

Besides the efficacy-related problems, ICBs induce the activation of T-cells and reduce the functions of regulatory T-cells (Tregs), which can lead to overstimulation of the immune system (Ramos-Casals et al., 2020). This can result in immunotherapy-related adverse events in all organs, including the gastrointestinal (GI) (e.g., colitis, hepatitis), dermatologic (e.g., alopecia, psoriasis, vitiligo), cardiovascular (e.g., myo- or endocarditis, cardiomyopathy), pulmonary (e.g., alveolitis, pneumonitis), neurologic (e.g., encephalitis, meningitis), systemic (e.g., cytokine release syndrome) and endocrine (e.g., thyroiditis, adrenitis) systems (Martins et al., 2019; Ramos-Casals et al., 2020; Chhabra and Kennedy, 2021).

This brief introduction highlights the importance of radiotherapy and immunotherapy as treatment options for CRC. However, it also shows that both treatments have drawbacks concerning inconsistency and side effects, with increasing evidence pointing towards the gut microbiome as a driver for these issues. In this review, we will focus on the gut microbiome and summarize how it influences radio- and immunotherapy efficacy and related side effects in CRC. Noteworthy, the efficacy and toxicity of chemotherapy, one of the most common treatment options for CRC (McQuade et al., 2017; Kumar et al., 2023), are also affected by the gut microbiome. We consider this out of scope and refer to extensive reviews (Kalasabail et al., 2021; Yu et al., 2022; Liu et al., 2023).

## 2 The gut microbiome in CRC

**Figure 1:**
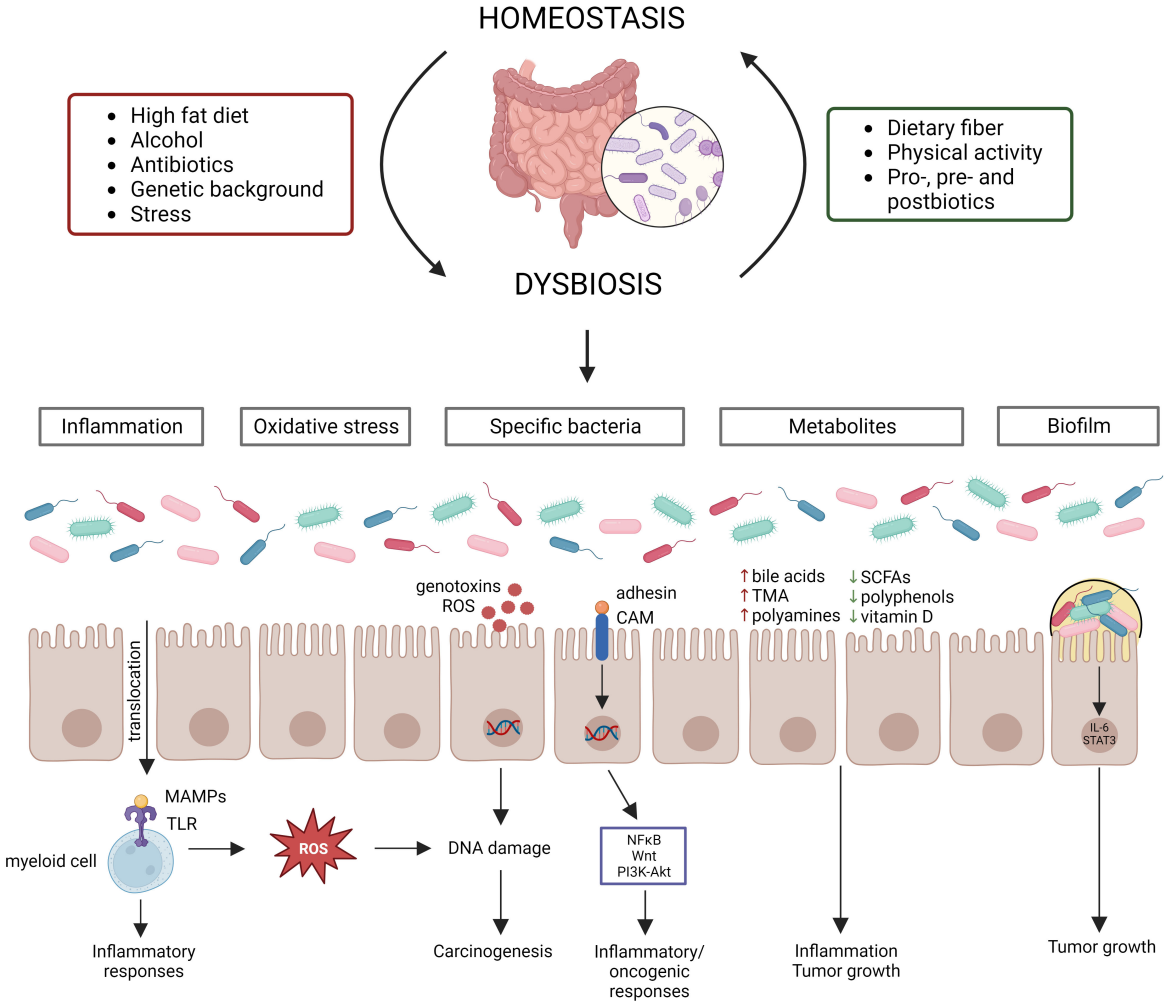
Gut microbiome homeostasis can be disrupted by various factors leading to dysbiosis, which contributes to CRC development via distinct mechanisms. Multiple factors influence the gut microbiome composition, establishing homeostasis or dysbiosis. Dysbiosis disrupts the intestinal barrier, resulting in translocation of bacteria and their products, which starts an inflammatory cascade. Moreover, certain bacteria produce reactive oxygen species (ROS) or genotoxins, while other bacteria activate inflammatory/oncogenic pathways. Chronic inflammation also results in the production of ROS, leading to DNA damage. Dysbiosis can also induce changes in metabolite levels, which contribute to inflammation and tumor growth. Lastly, biofilms can be formed due to dysbiosis, which also contribute to tumor growth.

A typical healthy gut microbiome contains trillions of microbes (Cresci and Bawden, 2015), covering between 300-1000 bacterial species, with *Bacteroidetes* and *Firmicutes* being the most abundant phyla (Lozupone et al., 2012). *Actinobacteria*, *Proteobacteria* and *Verrucomicrobia* are also present in relatively high abundances (Lozupone et al., 2012). The gut microbiome has three main functions: structural (maintenance of structural integrity of gut mucosal barrier), protective (co-relationship with the immune system to fight against invading microorganisms) and metabolic (e.g., participation in digestive processes, production of metabolites or other molecules) (Jandhyala et al., 2015; Rebersek, 2021). The host and its microbiome are one unit that co-evolve with each other and its composition can be influenced by multiple factors (e.g., diet, genetic background, stress, physical activity, and anti-, pro-, pre-, and postbiotic intake) (**Figure 1**) (Deschasaux et al., 2018; Leeming et al., 2019; Clooney et al., 2021). Disruption of the intestinal bacterial homeostasis is defined as dysbiosis, which is characterized by an altered diversity and abundance of the associated microbiota that can negatively impact the immune system (DeGruttola et al., 2016; Toor et al., 2019; Berg et al., 2020). Dysbiosis has also been linked to multiple diseases, such as neurological diseases, inflammatory diseases (e.g., inflammatory bowel disease (IBD)) and cancer (Hullar et al., 2014; Nishida et al., 2018; Sun and Shen, 2018).

Evidence from several studies has shown that dysbiosis can contribute to CRC carcinogenesis through multiple mechanisms, such as (1) pathogenic bacteria and their toxic products, (2) inflammation, (3) oxidative stress, (4) metabolites, and (5) biofilm formation (**Figure 1**). Dysbiosis is associated with a loss of protective bacteria, such as *Bifidobacterium animalis*, *Clostridium butyricum* and *Streptococcus thermophiles*, and an enrichment of cancer-promoting bacteria, such as *Fusobacterium nucleatum*, *Bacteroides fragilis*, *Escherichia coli*, *Streptococcus bovis*, *Enterococcus faecalis* and *Peptostreptococcus anaerobius* (Feng et al., 2015; de Almeida et al., 2018; Wassenaar, 2018; Long et al., 2019; Sun et al., 2019; Cheng et al., 2020; Deng et al., 2020; Li et al., 2021; Liu et al., 2021). It is to be noted that the contribution to CRC cannot be attributed to a sole bacterium, but multiple bacteria elicit negative effects that surpass those of the beneficial bacteria (Cheng et al., 2020). Several mechanisms by which these cancer-promoting bacteria can contribute to CRC development and progression have been proposed. For instance, *F. nucleatum* contains the surface virulence factors FadA, which activates inflammatory/oncogenic responses via NFκB and Wnt signaling, and Fap2, which protects CRC cells from immune attack (Sun et al., 2019). The *B. fragilis* toxin has also been shown to activate NFκB and Wnt signaling thereby promoting tumor cell proliferation and inducing metastasis (Cheng et al., 2020). The *E. coli* B2 phylotype can produce the genotoxin colibactin, which induces dsDNA breaks in the intestinal epithelial cells (Wassenaar, 2018). *S. bovis* has been shown to recruit CD11b^+^TLR4^+^ cells, which could promote a pro-tumor microenvironment (Deng et al., 2020). *E. faecalis* contributes to CRC carcinogenesis by producing reactive oxygen species (ROS), which induce DNA damage, and metalloproteases, which compromise barrier integrity and contribute to inflammation (de Almeida et al., 2018). *P. anaerobius* contains the surface protein PCWBR2, which preferentially interacts with CRC cells via α_2_/β_1_ integrin, after which the PI3K-Akt pathway is activated, eventually generating a pro-inflammatory response (Long et al., 2019). The detrimental effect of the CRC gut microbiome has been demonstrated by transplanting fecal samples from CRC patients to germ-free and conventional mice, leading to increased tumor proliferation, alteration of the gut microbiome, increased expression of pro-inflammatory genes and increased immune cell infiltration (Wong et al., 2017). The changed microbiome composition can affect the intestinal barrier, which results in translocation of microbiota and their products to extraintestinal sites, such as mesenteric lymph nodes, kidney, liver, spleen and bloodstream (Berg, 1999; Maisonneuve et al., 2018; Zou et al., 2018). Furthermore, bacterial translocation has been shown to contribute to CRC metastasis (Keramidaris et al., 2013). The translocated bacteria are recognized by immune cells via Toll-like receptors (TLRs), which induces the expression of cytokines, starting a pro-inflammatory cascade (Sánchez-Alcoholado et al., 2020). Inflammation induces DNA damage in intestinal cells, dysregulates anti-tumor immune responses and alters the gut microbiome composition, thereby contributing to CRC development (Nagao-Kitamoto et al., 2022). The idea that inflammation contributes to CRC carcinogenesis is supported by the observation that IBD patients have an increased risk of 10 – 15% to develop CRC (Loddo and Romano, 2015). Furthermore, dysbiosis is associated with altered levels of gut microbial metabolites, such as short-chain fatty acids (SCFAs), bile acids, trimethylamine (TMA), polyamines, polyphenols and vitamins (Bhat and Kapila, 2017; Zou et al., 2018). SCFAs, such as butyrate and propionate, can block inflammation by promoting Treg differentiation and stimulating macrophages and T-cells to produce IL-10 and TGF-β (Zou et al., 2018). The secondary bile acids deoxycholic acid and lithocholic acid promote CRC development by activating β-catenin and epidermal growth factor signaling and promoting cancer invasion and MAPK signaling, respectively (Baek et al., 2010; Ha and Park, 2010). TMA is further processed to trimethylamine-N-oxide, which is associated with CRC development, although its mechanism is still unclear (Xu et al., 2015). Polyamines are upregulated in CRC cells and stimulate tumor growth and immune evasion (Casero and Marton, 2007; Hayes et al., 2014; Novita Sari et al., 2021). Polyphenols show a protective effect by affecting inflammation, gut microbiota, epigenetics and mRNA expression (Ding et al., 2020). Vitamin D also elicits anticancer effects by influencing inflammation, apoptosis and Wnt/β-catenin signaling (Javed et al., 2020). The chronic inflammation induced by dysbiosis can result in the release of ROS, which can induce DNA damage of intestinal epithelial cells, eventually contributing to CRC development (Sorolla et al., 2021). Furthermore, it is hypothesized that certain bacteria can directly cause DNA damage (Artemev et al., 2022). For instance, *E. coli* has been shown to contribute to CRC development by downregulating DNA MMR (Sobhani et al., 2013). Dysbiosis can also contribute to the formation of biofilms, which have been linked to CRC development (Chew et al., 2020). Biofilms reduce the epithelial E-cadherin, resulting in disruption of the intestinal barrier, and stimulate IL-6/STAT3 signaling, inducing intestinal epithelial cell proliferation and tumor growth (Dejea et al., 2014; Johnson et al., 2015). In addition, biofilms can contribute to the formation of polyamine metabolites (Johnson et al., 2015).

### 2.1 The gut microbiome as a biomarker in CRC

Since the gut microbiome is altered in CRC patients, the gut microbiome is proposed as a screening, prognostic or predictive biomarker (Rebersek, 2021). For instance, it has already been shown that the gut microbiome differs between dMMR and pMMR cancer patients (Tahara et al., 2014; Mima et al., 2016; Gopalakrishnan et al., 2018; Hale et al., 2018; Matson et al., 2018; Routy et al., 2018; Xu et al., 2020; Kang et al., 2021; Jin et al., 2022). In one study, it was discovered that dMMR CRC patients showed a higher alpha diversity than pMMR CRC patients (Jin et al., 2022). Furthermore, dMMR CRC patients carried a higher abundance of the *Fusobacteria, Firmicutes, Verrucomicrobia* and *Actinobacteria* phyla and *Fusobacterium*, *Akkermansia*, *Bifidobacterium*, *Faecalibacterium*, *Streptococcus* and *Prevotella* genera. In contrast, pMMR CRC patients showed a higher abundance of *Proteobacteria* and more specifically the *Serratia*, *Cupriavidus* and *Sphingobium* genera. Based on the microbiome composition, Jin et al. predicted that the dMMR status could be associated with biosynthetic/metabolic pathways of glycan, vitamins and nucleotides, cell growth and death pathways, and genetic replication and repair pathways. The pMMR status was predicted to be associated with lipid, terpenoid, polyketone and amino acid metabolic pathways and membrane transport pathways. Other studies with CRC patients corroborated these observations and showed enrichment in *Fusobacteria* and *Bacteroidetes*, and reduction in *Firmicutes* and *Proteobacteria* in dMMR patients (Tahara et al., 2014; Mima et al., 2016; Hale et al., 2018; Xu et al., 2020; Kang et al., 2021). The pMMR patients also showed altered metabolic pathways of glycerol and phospholipid (Xu et al., 2020).

## 3 Radiotherapy-induced changes in the gut microbiome and its influence on toxicity

Despite its importance, the effect of irradiation on the human gut microbiome has been poorly studied (**Table 1**). An early study investigating the effect of pelvic radiotherapy on the gut microbiome in gynecological cancer patients discovered a decreased abundance of *E. coli*, *Aeromonas hydrophila*, *Peptococcus* spp., *Peptostreptococcus* spp., *F. nucleatum*, *Enterococcus faecium* and *Lactobacillus*, and an increased abundance of *Clostridium histolyticum*, *Clostridium bifermentans* and *Clostridium sporogenes* after radiotherapy (Cuzzolin et al., 1992). Other studies on gynecological cancer patients found that pelvic radiotherapy also decreased the abundance of the *Firmicutes* phylum and the *Lactobacillus* and *Bifidobacterium* genera, and increased the abundance of the *Fusobacterium* phylum and *Gammaproteobacteria*, *Bacilli* and *Negativicutes* classes (García-Peris et al., 2012; Nam et al., 2013; Ding et al., 2020). Another study investigated the effect of pelvic irradiation on the gut microbiome of 11 patients with different cancer types and discovered that irradiation induced an increase in *Bacteroides* and *Clostridium XIVa* and a decrease in *Lachnospiraceae, Faecalibacterium, Roseburia, Oscillibacter* and *Streptococcus* (Wang et al., 2015). When pediatric patients with rhabdomyosarcoma near the pelvic area received pelvic radiotherapy, the abundance of *Firmicutes* decreased whereas the abundance of *Proteobacteria*, *Actinobacteria* and *Bacteroidetes* increased (Sahly et al., 2019). Furthermore, the abundance of *Defluviitaleaceae, Ruminococcaceae, Clostridiales, Bacteroides, Streptococcus, Dorea, Bacteroides, Subdoligranulum, Escherichia-Shigella* increased after radiotherapy. When investigating the role of the gut microbiome on radiation-induced diarrhea in 10 patients with abdominal cancer, it was discovered that *Actinobacteria* and *Bacilli* were associated with radiation-induced diarrhea, whereas *Clostridia* was not (Manichanh et al., 2008). Before pelvic irradiation of 11 patients with different cancer types, patients who developed post-radiation diarrhea had increased abundances of *Bacteroides, Dialister, Veillonella* and decreased abundances of *Clostridium XI, Clostridium XVII, Faecalibacterium, Oscillibacter, Parabacteroides* and *Prevotella* in their fecal samples (Wang et al., 2015). Post-irradiation, the relative abundances of *Clostridium XI* together with *Alistipes, Bacteroides, Erysipelotrichaceae, Escherichia, Lachnospiracea* and *Megamonas*, were significantly higher in patients who developed diarrhea, whereas *Clostridium XIVa* and *Sutterella* were significantly lower. Reis Ferreira et al. investigated the effect of the gut microbiome on radiation enteropathy in prostate cancer patients and discovered that *Clostridium IV, Roseburia* and *Phascolarctobacterium* were associated with radiation enteropathy (Reis Ferreira et al., 2019). They also showed that homeostatic intestinal mucosa cytokines related to microbiota regulation and intestinal barrier maintenance were reduced in patients with radiation enteropathy. Cervical cancer patients with radiation enteritis (RE) showed a higher abundance of *Proteobacteria*, *Gammaproteobacteria*, *Megamonas, Novosphingobium* and *Prevotella* and a decreased abundance of *Bacteroides* compared to patients without RE (Wang et al., 2019). Furthermore, patients who would later develop RE showed a significantly higher abundance of *Coprococcus*, indicating that this could be a possible biomarker predicting the chance of RE.

**Table 1:** Radiation-induced up- or downregulation of bacteria and their correlation with radiotoxicity in cancer patients.

Abundance	GItoxicity	Bacteria	Cancer type	Place of irradiation	Type of irradiation	Dose	Sequencing technique	Reference
↑	/	*Dorea*	Rhabdomyosarcoma	Pelvis	Not specified	50.4 Gy	Illumina MiSeq	(Sahly et al., 2019)
↑	/	*Bacteroidetes*	Rhabdomyosarcoma	Pelvis	Not specified	50.4 Gy	Illumina MiSeq	(Sahly et al., 2019)
↑	/	*Fusobacterium*	Gynecological cancer	Pelvis	Not specified	50.4 Gy/day (5x/week for 5 weeks)	454 pyrosequencing	(Nam et al., 2013)
↑	/	*Clostridiales*	Rhabdomyosarcoma	Pelvis	Not specified	50.4 Gy	Illumina MiSeq	(Sahly et al., 2019)
↑	/	*Clostridium histolyticum*	Gynecological cancer	Pelvis	Not specified	40 Gy in 4-5 weeks	Culture counts/agar-based methods	(Cuzzolin et al., 1992)
↑	/	*Clostridium bifermentans*	Gynecological cancer	Pelvis	Not specified	40 Gy in 4-5 weeks	Culture counts/agar-based methods	(Cuzzolin et al., 1992)
↑	/	*Clostridium sporogenes*	Gynecological cancer	Pelvis	Not specified	40 Gy in 4-5 weeks	Culture counts/agar-based methods	(Cuzzolin et al., 1992)
↑	/	*Defluviitaleaceae*	Rhabdomyosarcoma	Pelvis	Not specified	50.4 Gy	Illumina MiSeq	(Sahly et al., 2019)
↑	/	*Ruminococcaceae*	Rhabdomyosarcoma	Pelvis	Not specified	50.4 Gy	Illumina MiSeq	(Sahly et al., 2019)
↑	/	*Subdoligranulum*	Rhabdomyosarcoma	Pelvis	Not specified	50.4 Gy	Illumina MiSeq	(Sahly et al., 2019)
↑	/	*Escherichia-shigella*	Rhabdomyosarcoma	Pelvis	Not specified	50.4 Gy	Illumina MiSeq	(Sahly et al., 2019)
↑	/	*Negativicutes*	Gynecological cancer	Pelvis	X-irradiation	41.8 – 50.4 Gy	Illumina MiSeq	(Ding et al., 2020)
↑	/	*Actinobacteria*	Rhabdomyosarcoma	Pelvis	Not specified	50.4 Gy	Illumina MiSeq	(Sahly et al., 2019)
↑	↓	*Clostridium XIVa*	Different types	Pelvis	Not specified	44-55 Gy	454 pyrosequencing	(Wang et al., 2015)
↑	/	*Bacilli*	Gynecological cancer	Pelvis	X-irradiation	48.1 – 50.4 Gy	Illumina MiSeq	(Ding et al., 2020)
/	↑		Abdominal cancer	Pelvis	Not specified	18-20 Gy/day (5x/week for 5 weeks)	PCR	(Manichanh et al., 2008)
↑	/	*Gammaproteobacteria*	Gynecological cancer	Pelvis	X-irradiation	41.8 – 50.4 Gy	Illumina MiSeq	(Ding et al., 2020)
/	↑		Cervical cancer	Pelvis	Not specified	50.4 Gy	Illumina HiSeq	(Wang et al., 2019)
↑	/	*Proteobacteria*	Rhabdomyosarcoma	Pelvis	Not specified	50.4 Gy	Illumina MiSeq	(Sahly et al., 2019)
/	↑		Cervical cancer	Pelvis	Not specified	50.4 Gy	Illumina HiSeq	(Wang et al., 2019)
↑	↑	*Bacteroides*	Different types	Pelvis	Not specified	44-55 Gy	454 pyrosequencing	(Wang et al., 2015)
	/		Rhabdomyosarcoma	Pelvis	Not specified	50.4 Gy	Illumina MiSeq	(Sahly et al., 2019)
/	↓		Cervical cancer	Pelvis	Not specified	50.4 Gy	Illumina HiSeq	(Wang et al., 2019)
↑	/	*Streptococcus*	Rhabdomyosarcoma	Pelvis	Not specified	50.4 Gy	Illumina MiSeq	(Sahly et al., 2019)
↓	/		Different types	Pelvis	Not specified	44-55 Gy	454 pyrosequencing	(Wang et al., 2015)
↓	/	*Firmicutes*	Gynecological cancer	Pelvis	Not specified	50.4 Gy/day (5x/week for 5 weeks)	454 pyrosequencing	(Nam et al., 2013)
			Rhabdomyosarcoma	Pelvis	Not specified	50.4 Gy	Illumina MiSeq	(Sahly et al., 2019)
↓	/	*Fusobacterium nucleatum*	Gynecological cancer	Pelvis	Not specified	40 Gy in 4-5 weeks	Culture counts/agar-based methods	(Cuzzolin et al., 1992)
↓	/	*Lactobacillus*	Gynecological cancer	Pelvis	Not specified	40 Gy in 4-5 weeks	Culture counts/agar-based methods	(Cuzzolin et al., 1992)
			Gynecological cancer	Pelvis	X-irradiation	52.2 Gy	Culture counts/FISH	(García-Peris et al., 2012)
↓	/	*Enterococcus faecium*	Gynecological cancer	Pelvis	Not specified	40 Gy in 4-5 weeks	Culture counts/agar-based methods	(Cuzzolin et al., 1992)
↓	/	*Peptococcus*	Gynecological cancer	Pelvis	Not specified	40 Gy in 4-5 weeks	Culture counts/agar-based methods	(Cuzzolin et al., 1992)
↓	/	*Peptostreptococcus*	Gynecological cancer	Pelvis	Not specified	40 Gy in 4-5 weeks	Culture counts/agar-based methods	(Cuzzolin et al., 1992)
↓	/	*Escherichia coli*	Gynecological cancer	Pelvis	Not specified	40 Gy in 4-5 weeks	Culture counts/agar-based methods	(Cuzzolin et al., 1992)
↓	/	*Bifidobacterium*	Gynecological cancer	Pelvis	X-irradiation	52.2 Gy	Culture counts/FISH	(García-Peris et al., 2012)
↓	/	*Aeromonas hydrophila*	Gynecological cancer	Pelvis	Not specified	40 Gy in 4-5 weeks	Culture counts/agar-based methods	(Cuzzolin et al., 1992)
↓	↓	*Faecalibacterium*	Different types	Pelvis	Not specified	44-55 Gy	454 pyrosequencing	(Wang et al., 2015)
↓	↓	*Oscillibacter*	Different types	Pelvis	Not specified	44-55 Gy	454 pyrosequencing	(Wang et al., 2015)
↓	↑	*Lachnospiraceae*	Different types	Pelvis	Not specified	44-55 Gy	454 pyrosequencing	(Wang et al., 2015)
↓	/	*Roseburia*	Different types	Pelvis	Not specified	44-55 Gy	454 pyrosequencing	(Wang et al., 2015)
/	↑		Prostate cancer	Pelvis/rectum	Not specified	Different doses/schemes	Illumina MiSeq	(Reis Ferreira et al., 2019)
/	↑	*Coprococcus*	Cervical cancer	Pelvis	Not specified	50.4 Gy	Illumina HiSeq	(Wang et al., 2019)
/	↑	*Clostridium IV*	Prostate cancer	Pelvis/rectum	Not specified	Different doses/schemes	Illumina MiSeq	(Reis Ferreira et al., 2019)
/	↑	*Erysipelotrichaceae*	Different types	Pelvis	Not specified	44-55 Gy	454 pyrosequencing	(Wang et al., 2015)
/	↑	*Dialister*	Different types	Pelvis	Not specified	44-55 Gy	454 pyrosequencing	(Wang et al., 2015)
/	↑	*Veillonella*	Different types	Pelvis	Not specified	44-55 Gy	454 pyrosequencing	(Wang et al., 2015)
/	↑	*Phascolarctobacterium*	Prostate cancer	Pelvis/rectum	Not specified	Different doses/schemes	Illumina MiSeq	(Reis Ferreira et al., 2019)
/	↑	*Megamonas*	Different types	Pelvis	Not specified	44-55 Gy	454 pyrosequencing	(Wang et al., 2015)
			Cervical cancer	Pelvis	Not specified	50.4 Gy	Illumina HiSeq	(Wang et al., 2019)
/	↑	*Alistipes*	Different types	Pelvis	Not specified	44-55 Gy	454 pyrosequencing	(Wang et al., 2015)
/	↑	*Escherichia*	Different types	Pelvis	Not specified	44-55 Gy	454 pyrosequencing	(Wang et al., 2015)
/	↑	*Actinomycetota*	Abdominal cancer	Pelvis	Not specified	18-20 Gy/day (5x/week for 5 weeks)	PCR	(Manichanh et al., 2008)
/	↑	*Novosphingobium*	Cervical cancer	Pelvis	Not specified	50.4 Gy	Illumina HiSeq	(Wang et al., 2019)
/	↓	*Clostridia*	Abdominal cancer	Pelvis	Not specified	18-20 Gy/day (5x/week for 5 weeks)	PCR	(Manichanh et al., 2008)
/	↓	*Clostridium XVII*	Different types	Pelvis	Not specified	44-55 Gy	454 pyrosequencing	(Wang et al., 2015)
/	↓	*Parabacteroides*	Different types	Pelvis	Not specified	44-55 Gy	454 pyrosequencing	(Wang et al., 2015)
/	↓	*Sutterella*	Different types	Pelvis	Not specified	44-55 Gy	454 pyrosequencing	(Wang et al., 2015)
/	↑/↓	*Clostridium XI*	Different types	Pelvis	Not specified	44-55 Gy	454 pyrosequencing	(Wang et al., 2015)
/	↓	*Prevotella*	Different types	Pelvis	Not specified	44-55 Gy	454 pyrosequencing	(Wang et al., 2015)
/	↑		Cervical cancer	Pelvis	Not specified	50.4 Gy	Illumina HiSeq	(Wang et al., 2019)

↑, increase; ↓, decrease; ↑/↓, increase and decrease.

The effect of irradiation on the gut microbiome has been more extensively investigated in mice, although only in healthy mice (**Table 2**). Acute pelvic irradiation of mice induced intestinal damage and inflammation, which resulted in loss of tight junctions, eventually leading to translocation of bacteria to mesenteric lymph nodes and dysbiosis (Segers et al., 2021). Radiation-induced dysbiosis can aggravate intestinal inflammation, which was shown by an increased expression of IL-1β (Gerassy-Vainberg et al., 2018). Members of the *Ruminococcaceae*, *Lachnospiraceae* and *Porhyromonadaceae* families could be identified as markers of dysbiosis (Segers et al., 2021). Gamma irradiation of mice led to increased proportions of *Alistipes, Lactobacillus* and *Akkermansia*, but reduced proportions of *Barnesiella, Prevotella, Bacteroides, Oscillibacter, Pseudoflavonifractor* and *Mucispirillum* in the large intestine (Kim et al., 2015). In the small intestine, irradiation led to an increase in *Corynebacterium* and decrease in *Alistipes*. In addition, Gerassy-Vainberg et al. exposed mice to rectal irradiation, leading to an increased abundance of *Proteobacteria* and *Verrucomicrobia* and a decreased abundance of *Firmicutes* (Gerassy-Vainberg et al., 2018). When exposed to low-dose irradiation, mice showed an increased abundance of *Clostridium, Helicobacter* and *Oscillibacter* and a decreased abundance of *Bacteroides* and *Barnesiella* (Liu et al., 2019). The latter study also showed that irradiated mice had perturbed metabolite levels (e.g., downregulation of glucose, pyruvic acid, pinitol, and upregulation of hydroquinone, octadecanol and O-phosphoserine), which were predicted to be involved in glucagon signaling, central carbon metabolism and type II diabetes. Goudarzi et al. investigated the effect of X-ray radiation on mice and discovered that irradiation led to an increased abundance of *Lactobacillaceae* and *Staphylococcaceae*, and a decreased abundance of *Lachnospiraceae*, *Ruminococcaceae* and *Clostridiaceae* (Goudarzi et al., 2016). The metabolomic data revealed statistically significant changes in the microbiota-derived products, such as pipecolic acid, glutaconic acid, urobilinogen and homogentisic acid. In addition, significant changes were detected in bile acids (e.g., taurocholic acid and 12-ketodeoxycholic acid), which may be associated with an altered abundance of *Ruminococcus gnavus* that is able to transform bile acids (Devlin and Fischbach, 2015; Goudarzi et al., 2016). An increased abundance of *Bacteroidia* and a decreased abundance of *Clostridia* could still be observed in irradiated mice ten months after exposure, advocating long-term effects of irradiation (Zhao et al., 2019). The gut microbiome can also influence the risk of side effects, corroborated by the higher survival rates of mice that received antibiotics before radiotherapy (Cui et al., 2017). Members of *Akkermansia, Bacteroides, Parabacteroides, Sutterella* and *Turicibacter* were more abundant in mice who developed radiation proctitis (Gerassy-Vainberg et al., 2018). Furthermore, irradiation of germ-free (GF) mice that were inoculated with fecal material from previously irradiated mice led to worse irradiation-induced damage compared to irradiation of GF mice that were inoculated with fecal material from naïve mice (Gerassy-Vainberg et al., 2018).

**Table 2:** Radiation-induced up- or downregulation of bacteria and their correlation with radiotoxicity in healthy mice.

Abundance	GI toxicity	Bacteria	Mouse strain	Place of irradiation	Type of irradiation	Dose	Sequencing technique	Reference
↑	/	*Clostridium*	BALB/c	Whole body	Co60 γ-irradiation	0.5 Gy	Illumina HiSeq	(Liu et al., 2019)
↑	/	*Lactobacillaceae*	C57Bl/6	Whole body	X-irradiation	5 or 12 Gy	Illumina HiSeq	(Goudarzi et al., 2016)
↑	/	*Staphylococcaceae*	C57Bl/6	Whole body	X-irradiation	5 or 12 Gy	Illumina HiSeq	(Goudarzi et al., 2016)
↑	/	*Bacteroidia*	C57Bl/6	Whole body	Cs137 γ-irradiation	8 Gy	Illumina HiSeq	(Zhao et al., 2019)
↑	/	*Verrucomicrobia*	C57Bl/6	Rectum	Co60 γ-irradiation	22 Gy (5.5 Gy/day for 4 days)	Illumina MiSeq	(Gerassy-Vainberg et al., 2018)
↑	/	*Corynebacterium*	C57Bl/6	Not specified	Co60 γ-irradiation	8 Gy	Illumina MiSeq	(Kim et al., 2015)
↑	/	*Helicobacter*	BALB/c	Whole body	Co60 γ-irradiation	0.5 Gy	Illumina HiSeq	(Liu et al., 2019)
↑	/	*Proteobacteria*	C57Bl/6	Rectum	Co60 γ-irradiation	22 Gy (5.5 Gy/day for 4 days)	Illumina MiSeq	(Gerassy-Vainberg et al., 2018)
↓	/	*Oscillibacter*	C57Bl/6 BALB/c	Not specified	Co60 γ-irradiation	8 Gy	Illumina MiSeq	(Kim et al., 2015)
↑	/		BALB/c	Whole body	Co60 γ-irradiation	0.5 Gy	Illumina HiSeq	(Liu et al., 2019)
↑	/	*Lactobacillus*	C57Bl/6	Not specified	Co60 γ-irradiation	8 Gy	Illumina MiSeq	(Kim et al., 2015)
↓	/		C57Bl/6	Whole body	Cs137 γ-irradiation	6,5 Gy	Illumina HiSeq	(Cui et al., 2017)
↑/↓	/	*Alistipes*	C57Bl/6	Not specified	Co60 γ-irradiation	8 Gy	Illumina MiSeq	(Kim et al., 2015)
↑	/	*Akkermansia*	C57Bl/6	Not specified	Co60 γ-irradiation	8 Gy	Illumina MiSeq	(Kim et al., 2015)
/	↑		C57Bl/6	Rectum	Co60 γ-irradiation	22 Gy (5.5 Gy/day for 4 days)	Illumina MiSeq	(Gerassy-Vainberg et al., 2018)
↑	↑	*Sutterella*	C57Bl/6	Rectum	Co60 γ-irradiation	Not specified	Illumina MiSeq	(Gerassy-Vainberg et al., 2018)
↓	/	*Firmicutes*	C57Bl/6	Rectum	Co60 γ-irradiation	Not specified	Illumina MiSeq	(Gerassy-Vainberg et al., 2018)
↓	/	*Pseudoflavonifractor*	C57Bl/6	Not specified	Co60 γ-irradiation	8 Gy	Illumina MiSeq	(Kim et al., 2015)
↓	/	*Lachnospiraceae*	C57Bl/6	Whole body	X-irradiation	5 or 12 Gy	Illumina HiSeq	(Goudarzi et al., 2016)
↓	/	*Clostridia*	C57Bl/6	Whole body	Cs137 γ-irradiation	8 Gy	Illumina HiSeq	(Zhao et al., 2019)
↓	/	*Clostridiaceae*	C57Bl/6	Whole body	X-irradiation	5 or 12 Gy	Illumina HiSeq	(Goudarzi et al., 2016)
↓	/	*Prevotella*	C57Bl/6	Not specified	Co60 γ-irradiation	8 Gy	Illumina MiSeq	(Kim et al., 2015)
↓	/	*Barnesiella*	C57Bl/6	Not specified	Co60 γ-irradiation	8 Gy	Illumina MiSeq	(Kim et al., 2015)
			BALB/c	Whole body	Co60 γ-irradiation	0.5 Gy	Illumina HiSeq	(Liu et al., 2019)
↓	/	*Mucispirillum*	C57Bl/6	Not specified	Co60 γ-irradiation	8 Gy	Illumina MiSeq	(Kim et al., 2015)
↓	/	*Ruminococcaceae*	C57Bl/6	Whole body	X-irradiation	5 or 12 Gy	Illumina HiSeq	(Goudarzi et al., 2016)
↓	/	*Bacteroides*	C57Bl/6	Not specified	Co60 γ-irradiation	8 Gy	Illumina MiSeq	(Kim et al., 2015)
			BALB/c	Whole body	Co60 γ-irradiation	0.5 Gy	Illumina HiSeq	(Liu et al., 2019)
			C57Bl/6	Whole body	Cs137 γ-irradiation	6.5 Gy	Illumina HiSeq	(Cui et al., 2017)
/	↑		C57Bl/6	Rectum	Co60 γ-irradiation	22 Gy (5.5 Gy/day for 4 days)	Illumina MiSeq	(Gerassy-Vainberg et al., 2018)
/	↑	*Turicibacter*	C57Bl/6	Rectum	Co60 γ-irradiation	22 Gy (5.5 Gy/day for 4 days)	Illumina MiSeq	(Gerassy-Vainberg et al., 2018)
/	↑	*Parabacteroides*	C57Bl/6	Rectum	Co60 γ-irradiation	22 Gy (5.5 Gy/day for 4 days)	Illumina MiSeq	(Gerassy-Vainberg et al., 2018)

↑, increase; ↓, decrease; ↑/↓, increase and decrease.

It is proposed that the gut microbiome can influence radiotherapy response, for which some markers have already been defined (Liu et al., 2021). Autophagy has been shown to be related to radiosensitivity/radioresistance of the tumor. Inhibition of autophagy induces radioresistance whereas induction of autophagy induces radiosensitivity (Kuwahara et al., 2011). *Fusobacterium nucleatum* has been shown to activate autophagy, leading to chemoresistance (Yu et al., 2017). Up until now, no studies have shown the effect of the gut microbiome on radioresistance via autophagy. Furthermore, it has been shown that other factors such as the time when radiation is given can affect the gut microbiome leading to differences in radiosensitivity (Cui et al., 2016; Chan et al., 2017). Fasting-induced adipose factor (FIAF) is a microbiota-regulated protein that has been related to the radiosensitivity of endothelial cells and lymphocytes, and could be used as a protector for radiotoxicity (Crawford and Gordon, 2005). *Bacteroides thetaiotaomicron* and *Enterococcus faecalis* increase the FIAF production, whereas *Escherichia coli* decreases the FIAF production (Grootaert et al., 2011). However, there is currently no information available about the influence of the gut microbiome on radiotherapy efficacy as a stand-alone treatment.

**Figure 2:**
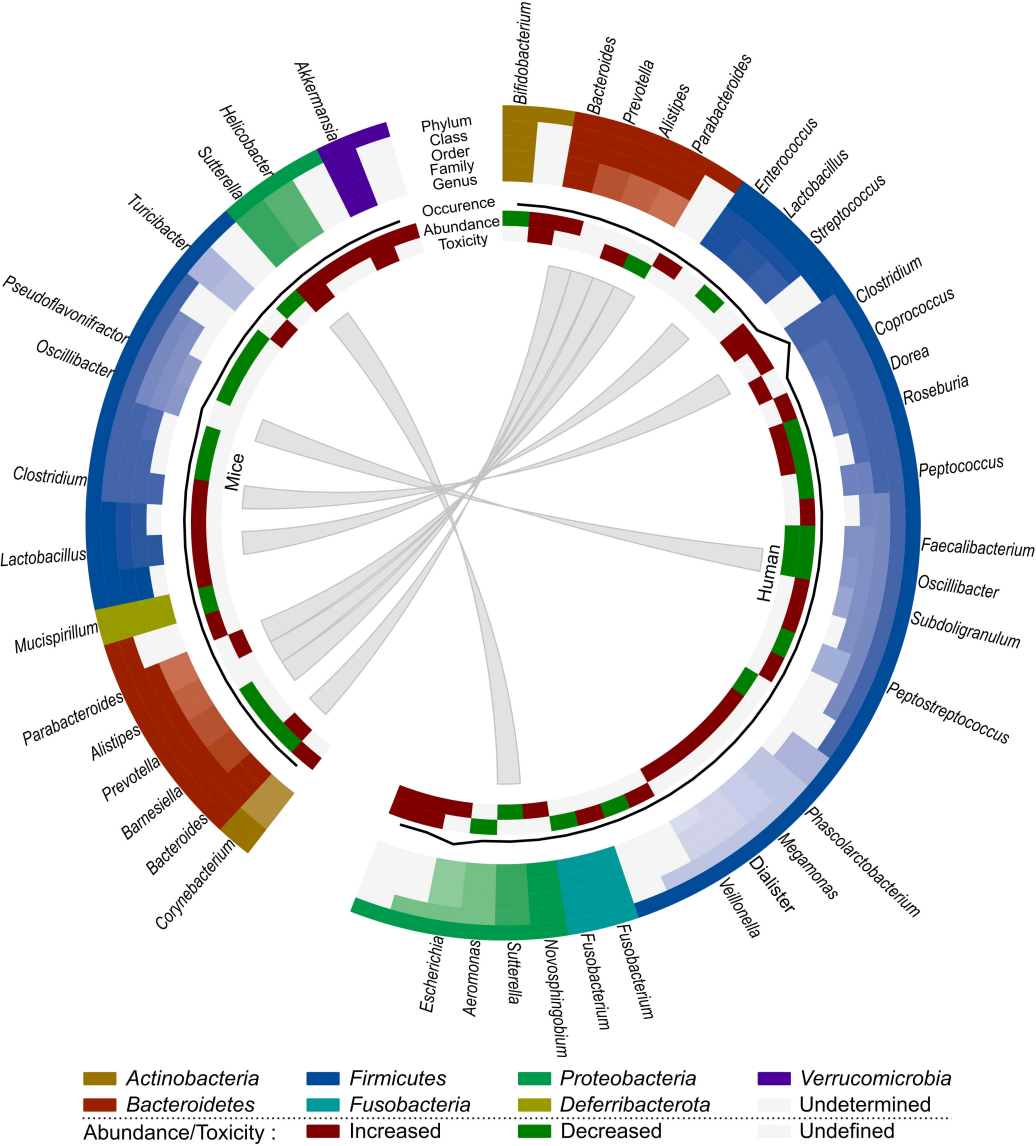
Comparison of the effect of radiotherapy on the gut microbiome of mice and humans. Each distinct phylum is represented by a separate color, as are distinct taxonomic classes, orders, families and genera. Similar observations are connected. Additional information is provided for each observation, including its occurrence in literature (line, scale maximum is 7), (relative) abundance of member and toxicity (increased: red; decreased: green; undefined: grey).

We have opted to list the outcome of different studies exhaustively in order to highlight the complex effects of irradiation on the gut microbiome. Based on all these observations, we can conclude that the presence of *Bacilli, Negativicutes, Lachnospiraceae, Coprococcus, Escherichia* and *Alistipes* seem to be related to radiation-induced toxicity in human cancer patients, whereas *Akkermansia*, *Bacteroides*, *Sutterella*, *Parabacteroides* and *Turicibacter* are associated with radiation-induced toxicity in mice (**Figure 2**). However, *Sutterella* and *Parabacteroides* seem to be associated with less toxicity in cancer patients. In addition, *Faecalibacterium* and *Oscillibacter* reduce toxicity and the contribution of *Bacteroides* remains unclear. Nevertheless, comparison of mice and human data is difficult, since besides intrinsic differences in their microbiome, there is a lack of data about the role of the microbiome in radiotherapy-induced toxicity in cancer-bearing mice. Moreover, the impact of the gut microbiome on radiotherapy efficacy remains unknown in both human and mice, presenting an intriguing area for further investigation.

## 4 The influence of the gut microbiome on anti-tumor responses and immunotherapy efficacy

**Figure 3:**
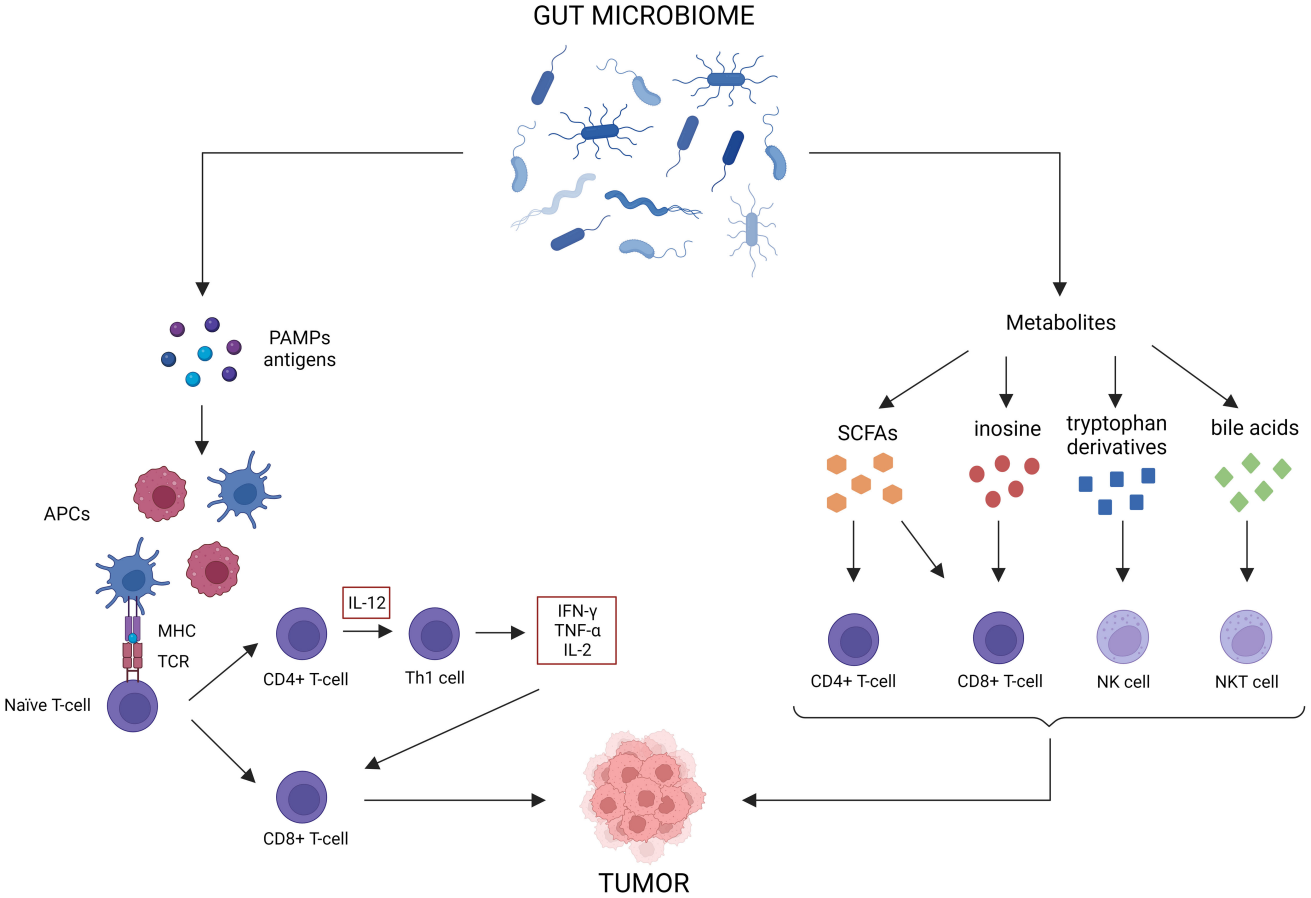
Antitumor immune effects of the gut microbiome. The gut microbiome possesses multiple immune-related antitumor mechanisms. First of all, certain microorganisms release pattern-associated molecular patterns (PAMPs), which activate antigen-presenting cells (APCs) to stimulate CD4^+^ and CD8^+^ T-cell responses. Molecular mimicry between tumor and microbial antigens can contribute to these responses as well. Lastly, gut microbiome metabolites, such as short-chain fatty acids (SCFAs), inosine, tryptophan derivatives and bile acids, can activate antitumor immune cells.

For CRC, ICBs such as anti-PD-L1, anti-PD-1 or anti-CTLA-4, are only indicated for the treatment of dMMR cancer (Ganesh et al., 2019). However, only 15% of all CRC patients are dMMR, meaning only a small subset of patients will benefit from immunotherapy as a monotherapy (Boland et al., 1998). Most patients will need additional treatment to achieve an optimal response. As the gut microbiome is involved in maturation of both the innate and the adaptive immune system, perturbation can result in aberrant immune responses that can contribute to multiple GI disorders, such as IBD and cancer (Zheng et al., 2020; Jain et al., 2021). Likewise, the abundance of some bacteria (e.g., *Bacteroidetes*, *Akkermansia*, *Lactobacillus*) are positively correlated to antitumor immunity, whereas others are negatively correlated (e.g., *Firmicutes*, *Proteobacteria*, *Parabacteroides*) (Xia et al., 2020). The gut microbiome can elicit antitumor immune responses via different mechanisms (**Figure 3**) (Park et al., 2020). For instance, some microorganisms contain pathogen-associated molecular patterns that can activate antigen-presenting cells (APCs) via pattern recognition receptors (Panda et al., 2018). This induces the activation of CD4^+^ and CD8^+^ T-cells, which influences cytokine expression (TNF-α, IFN-γ, IL-2) and stimulates tumor cell killing (Li et al., 2019). Microbial and tumor cell products may also share antigen sequences, leading to T-cell cross-reactivity between these antigens, which induces antigen-specific immune responses known as molecular mimicry (Baruch et al., 2021). Furthermore, multiple gut microbiota metabolites can elicit antitumor effects. For instance, SCFAs have been shown to reduce colon liver metastases in mice models, activate cytotoxic CD8^+^ T-cells and their memory potential and stimulate and differentiate CD4^+^ T-cells (Park et al., 2016; Bachem et al., 2019; Ma et al., 2020; He et al., 2021). Inosine has been shown to act as a carbon source for CD8^+^ T-cells and stimulates T-cell proliferation and differentiation while enhancing sensitivity to ICBs (Wang et al., 2020). Tryptophan derivatives can stimulate the cytolytic activity of natural killer (NK) cells (Shin et al., 2013). Finally, the gut microbiome can also modify bile acids, which are able to activate antitumor immune cells, such as natural killer T-cells (NKT) (Sipe et al., 2020).

Although there is a lot of knowledge on the influence of the gut microbiome on immunotherapy efficacy for several cancer types, there is limited information for CRC, which is surprising considering its involvement with the microbiome (**Table 3**). A study involving a cohort of GI cancer patients, showed that the *Prevotella/Bacteroides* ratio was higher in anti-PD-1 responders (Peng et al., 2020). Anti-PD-1 responders showed an increased abundance of *Lachnoclostridium*, *Parabacteroides*, *Lachnospiraceae*, *Ruminococcaceae*, *Flavonifractor* and *Dialister*, whereas non-responders showed an increased abundance of *Bacteroides*, *Parabacteroides*, *Coprococcus* and *Subdoligranulum*. SCFA-producing bacteria, such as *Eubacterium*, *Lactobacillus* and *Streptococcus* were associated with better anti-PD-1 efficacy for GI cancers in general.

**Table 3:** Bacteria and their correlation with ICB efficacy in CRC mice and GI cancer patients.

Bacteria	Effect on ICB efficacy	Type of ICB	Dose	ICB frequency	Mice/human	Reference
*Ruminococcaceae*	Positive	Anti-PD-1	Not specified	Every 2/3 weeks	Human	(Peng et al., 2020)
*Lachnospiraceae*	Positive	Anti-PD-1	Not specified	Every 2/3 weeks	Human	(Peng et al., 2020)
*Lachnoclostridium*	Positive	Anti-PD-1	Not specified	Every 2/3 weeks	Human	(Peng et al., 2020)
*Flavonifractor*	Positive	Anti-PD-1	Not specified	Every 2/3 weeks	Human	(Peng et al., 2020)
*Dialister*	Positive	Anti-PD-1	Not specified	Every 2/3 weeks	Human	(Peng et al., 2020)
*Subdoligranulum*	Negative	Anti-PD-1	Not specified	Every 2/3 weeks	Human	(Peng et al., 2020)
*Coprococcus*	Negative	Anti-PD-1	Not specified	Every 2/3 weeks	Human	(Peng et al., 2020)
*Parabacteroides*	Positive/negative	Anti-PD-1	Not specified	Every 2/3 weeks	Human	(Peng et al., 2020)
*Bacteroides*	Negative	Anti-PD-1	Not specified	Every 2/3 weeks	Human	(Peng et al., 2020)
	Negative	Anti-PD-1	250 µg	Every 3 days for a total of 5 injections	Mice (BALB/c)	(Xu et al., 2020)
*Bacteroides_sp._CAG:927*	Negative	Anti-PD-1	250 µg	Every 3 days for a total of 5 injections	Mice (BALB/c)	(Xu et al., 2020)
*Prevotella_sp._CAG:1031*	Positive	Anti-PD-1	250 µg	Every 3 days for a total of 5 injections	Mice (BALB/c)	(Xu et al., 2020)
*Akkermansia muciniphila*	Positive	Anti-PD-1	250 µg	Every 3 days for a total of 5 injections	Mice (BALB/c)	(Xu et al., 2020)
*Bifidobacterium pseudolongum*	Positive	Anti-CTLA-4	100 µg	Every 3 days for a total of 5 injections	Mice (C57Bl/6J)	(Mager et al., 2020)
*Olsenella*	Positive	Anti-CTLA-4	100 µg	Every 3 days for a total of 5 injections	Mice (C57Bl/6J)	(Mager et al., 2020)
*Lactobacillus johnsonii*	Positive	Anti-CTLA-4	100 µg	Every 3 days for a total of 5 injections	Mice (C57Bl/6J)	(Mager et al., 2020)
*Lactobacillus acidophilus*	Positive	Anti-CTLA-4	50 µg	Every other day for a total of 17 injections	Mice (BALB/c)	(Zhuo et al., 2019)
*Parabacteroides distasonis*	Positive	Anti-PD-1 Anti-CTLA-4	200 µg	Every 3 days for a total of 3 injections	Mice (C57Bl/6J)	(Tanoue et al., 2019)
*Parabacteroides gordonii*	Positive	Anti-PD-1 Anti-CTLA-4	200 µg	Every 3 days for a total of 3 injections	Mice (C57Bl/6J)	(Tanoue et al., 2019)
*Alistipes senegalensis*	Positive	Anti-PD-1 Anti-CTLA-4	200 µg	Every 3 days for a total of 3 injections	Mice (C57Bl/6J)	(Tanoue et al., 2019)
*Parabacteroides johnsonii*	Positive	Anti-PD-1 Anti-CTLA-4	200 µg	Every 3 days for a total of 3 injections	Mice (C57Bl/6J)	(Tanoue et al., 2019)
*Paraprevotella xylaniphila*	Positive	Anti-PD-1 Anti-CTLA-4	200 µg	Every 3 days for a total of 3 injections	Mice (C57Bl/6J)	(Tanoue et al., 2019)
*Bacteroides dorei*	Positive	Anti-PD-1 Anti-CTLA-4	200 µg	Every 3 days for a total of 3 injections	Mice (C57Bl/6J)	(Tanoue et al., 2019)
*Bacteroides uniformis JCM 5828*	Positive	Anti-PD-1 Anti-CTLA-4	200 µg	Every 3 days for a total of 3 injections	Mice (C57Bl/6J)	(Tanoue et al., 2019)
*Eubacterium limosum*	Positive	Anti-PD-1 Anti-CTLA-4	200 µg	Every 3 days for a total of 3 injections	Mice (C57Bl/6J)	(Tanoue et al., 2019)
*Ruminococcaceae bacterium cv2*	Positive	Anti-PD-1 Anti-CTLA-4	200 µg	Every 3 days for a total of 3 injections	Mice (C57Bl/6J)	(Tanoue et al., 2019)
*Phascolarctobacterium faecium*	Positive	Anti-PD-1 Anti-CTLA-4	200 µg	Every 3 days for a total of 3 injections	Mice (C57Bl/6J)	(Tanoue et al., 2019)
*Fusobacterium ulcerans*	Positive	Anti-PD-1 Anti-CTLA-4	200 µg	Every 3 days for a total of 3 injections	Mice (C57Bl/6J)	(Tanoue et al., 2019)

In CRC mice, it was shown that treatment with antibiotics and anti-PD-1 resulted in higher tumor volumes, indicating that a homeostatic gut microbiome is needed to achieve an optimal anti-PD-1 response (Xu et al., 2020). Furthermore, *Prevotella* sp. CAG:1031 and *Akkermansia muciniphila* were related to a better anti-PD-1 response, whereas *Bacteroides* and *Bacteroides* sp. CAG:927 appeared to be related to a poorer anti-PD-1 response. A significant upregulation of the glycerophospholipid metabolism could be observed in anti-PD-1 responders. This led the authors to speculate that differences in the microbiome affect the glycerophospholipid metabolism, which alters IFN-γ and IL-2 expression in the tumor microenvironment, resulting in better anti-PD-1 response. When fecal samples from healthy volunteers were administered to GF mice, the extent of IFN-γ^+^ CD8^+^ T-cell induction was donor dependent (Tanoue et al., 2019). Eleven species were positively associated with IFN-γ^+^ CD8^+^ T-cell induction: *Parabacteroides distasonis, Parabacteroides gordonii, Alistipes senegalensis, Parabacteroides johnsonii, Paraprevotella xylaniphila, Bacteroides dorei, Bacteroides uniformis* JCM 5828*, Eubacterium limosum, Ruminococcaceae* bacterium cv2*, Phascolarctobacterium faecium* and *Fusobacterium ulcerans*. Furthermore, MC38-tumor bearing mice showed a better response to anti-PD-1 and anti-CTLA-4 treatment after engraftment with these eleven strains. Treatment of the more physiological AOM/DSS CRC mouse model with anti-CTLA-4 or anti-PD-L1 led to smaller and fewer tumors, reduced cancer stem cells, increased immune cell infiltration into tumors, increased CD8^+^ T-cell frequencies in tumor draining lymph nodes and increased splenic CD4^+^ and CD8^+^ T-cell activation (Mager et al., 2020). The metabolite inosine seemed to be responsible for enhancing ICB efficacy. At microbiome level, *Bifidobacterium pseudolongum, Olsenella* sp.*, Colidextribacter* sp.*, Bacillus thermoamylovorans, Prevotella* sp.*, Lactobacillus reuteri, Akkermansia muciniphila* could only be found in ICB-treated tumors, whereas *Collinsella* sp.*, Clostridium cocleatum* and *Bacteroides* sp. were exclusively found in control-treated tumors. Afterwards, it was shown that *Bifidobacterium pseudolongum* and *Olsenella* sp. were associated with better ICB-efficacy. Another study on AOM/DSS CRC mice discovered that treatment with anti-CTLA-4 and lysates of *Lactobacillus acidophilus* reduced the amount of tumors and Tregs and M2 macrophages in mesenteric lymph nodes and increased the amount of IL-2 and IFN-γ in the serum, and CD8^+^ T-cell infiltration in the tumor (Zhuo et al., 2019). Furthermore, combined treatment with anti-CTLA-4 and *Lacobacillus acidophilus* lysates restored the dysregulated CRC microbiome by reducing the abundance of *Proteobacteria* that was increased after tumor development.

**Figure 4:**
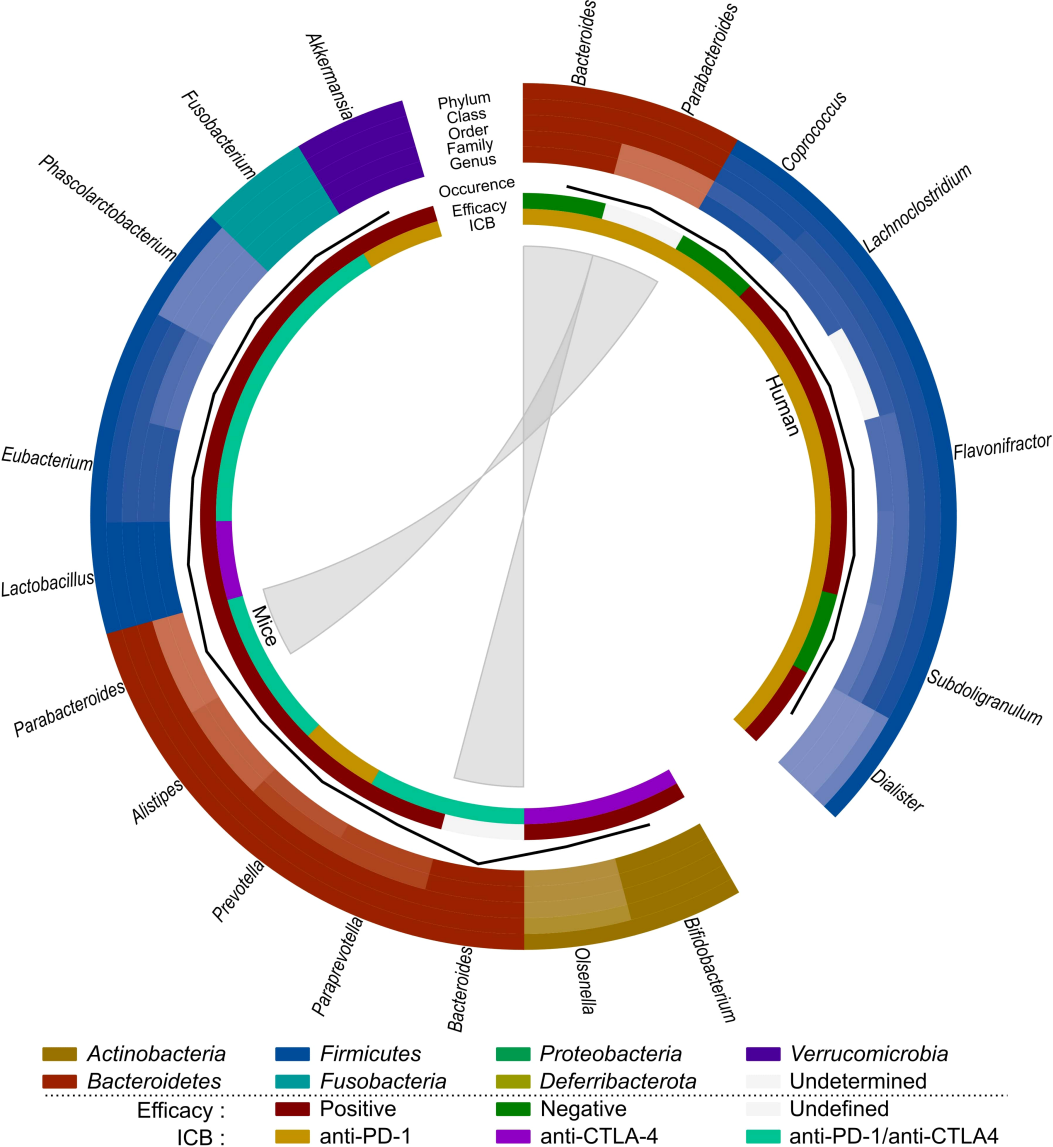
Comparison of the effect of immunotherapy on the gut microbiome of mice and humans. Each distinct phylum is represented by a separate color, as are distinct taxonomic classes, orders, families, and genera. Similar observations are connected. Additional information is provided for each observation, including its occurrence in literature (line, scale maximum is 4), impact on efficacy (increased: red; decreased: green; undefined: grey) and ICB studied (anti-PD-1: yellow; anti-CTLA-4: purple; anti-PD-1/anti-CTLA-4: teal).

Based on these observations (**Table 3**; **Figure 4**), SCFA-producing bacteria, such as *Lactobacillus* and *Eubacterium*, seem to enhance immunotherapy efficacy in both mice and humans. *Parabacteroides* and *Akkermansia* also seem to be associated with better immunotherapy efficacy in mice, which contradicts their previously mentioned role in radiation-induced toxicity. *Bacteroides* seems to be negatively related to immunotherapy efficacy in humans, but its impact on immunotherapeutic efficacy in mice is unclear and requires further investigation. As *Coprococcus* is associated with radiation-induced toxicity as well as worse ICB efficacy, it is a potential detrimental genus for both treatments. Finally, since the role of the gut microbiome on immune-related adverse events has not yet been investigated for CRC, this is an interesting subject for prospective research.

## 5 Combined radio- and immunotherapy treatment

The view of radiotherapy as a simple cytotoxic agent has dramatically changed in recent years. It is now accepted that radiotherapy can reshape the tumor microenvironment by modulating the immune response (Frey et al., 2017). Therefore, there is a rationale to use immunotherapy together with radiotherapy to boost therapeutic outcomes. However, there is still a need to define biomarkers to identify patients who would benefit most from dual radio- and immunotherapy, optimize optimal sequences/schedules for combined radio- and immunotherapy and identify mechanisms to overcome resistance (Mondini et al., 2020). Furthermore, the effects of dual radio- and immunotherapy on healthy tissues and related toxicity remain largely unknown.

### 5.1 Immune activating and suppressing effects of radiotherapy

**Figure 5:**
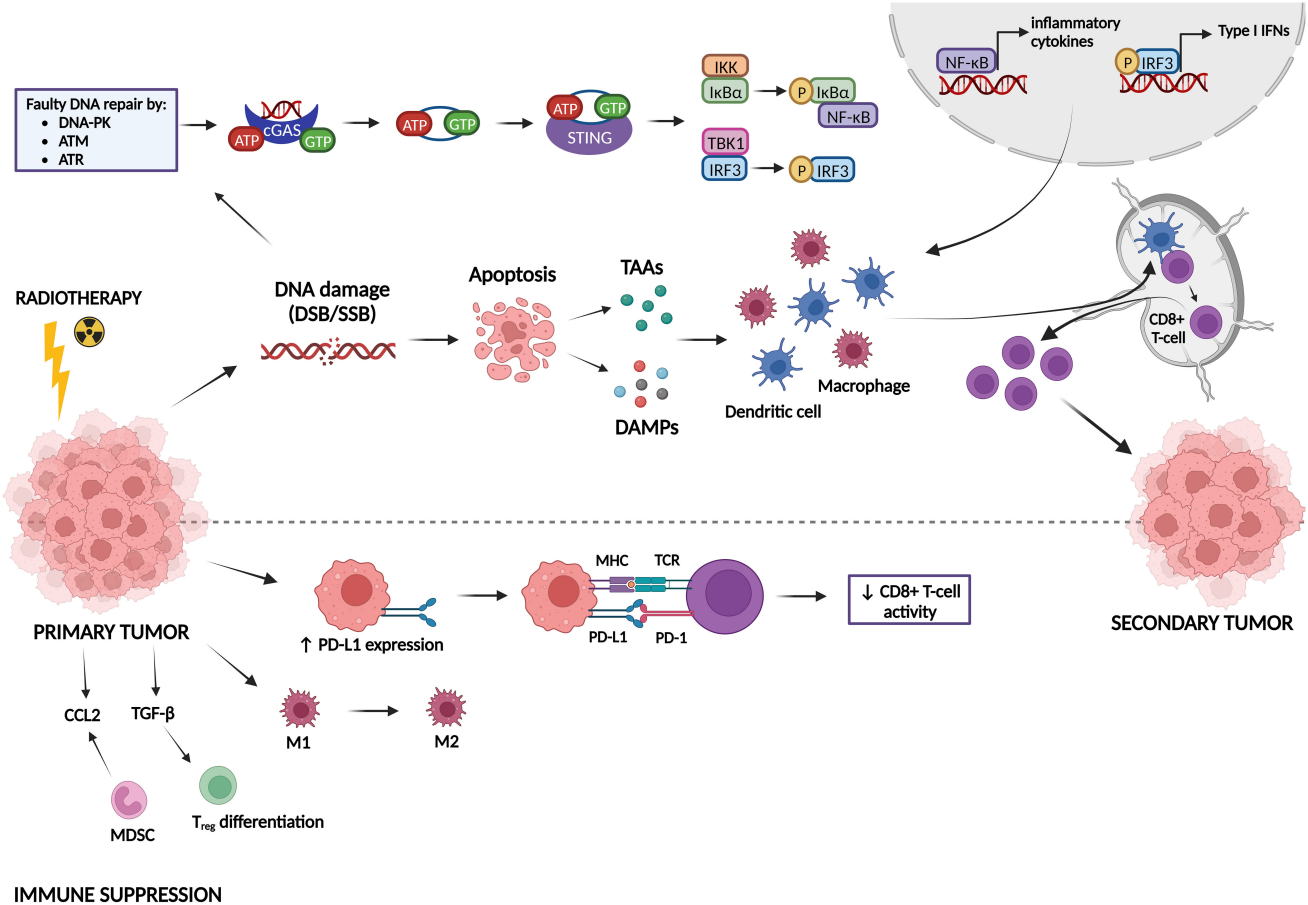
Immune activating and suppressing effects of radiotherapy. Radiotherapy induces DNA damage, which can lead either to DNA repair or to apoptosis. Faulty repaired DNA stimulates the cyclic GMP-AMP synthase (cGAS)-stimulator of IFN genes (STING) pathway, leading to the transcription of type I IFN genes and other inflammatory cytokines. In addition, apoptotic cells release tumor-associated antigens (TAAs) and damage-associated molecular patterns (DAMPs). All these factors can activate dendritic cells (DCs) and macrophages, which stimulate CD8^+^ T-cell priming, inducing immunogenic responses against a secondary tumor. On the other hand, radiotherapy can also act in an immunosuppressive manner. It induces upregulation of PD-L1 expression on tumor cells, leading to inhibition of CD8^+^ T-cell activation. Furthermore, radiotherapy can promote differentiation/attraction of immunosuppressive immune cells.

Radiotherapy can induce both immune-activating and immune-suppressing effects, which are summarized in **Figure 5**. Radiotherapy can promote cancer cell killing by inducing DNA damage, but can also activate the immune system, which can be observed by the induction of the abscopal effect and immunogenic cell death (ICD) (Golden and Apetoh, 2015; Brix et al., 2017; Wang et al., 2018). The abscopal effect, which is defined as tumor responses at sites distant from the irradiated site, was first observed by Dr. R. H. Mole in 1953 (Mole, 1953). However, abscopal effects after radiotherapy are rarely observed in the clinic because it is hard to induce an immune response in non-irradiated metastases that are characterized by a specific microenvironment and because metastases might be antigenically heterogeneous (Vanpouille-Box et al., 2018). Radiotherapy can induce the abscopal effect by activation of CD8^+^ T-cells by antigen-presenting cells (APCs) that have captured radiation-released tumor-associated antigens (TAAs) (Baba et al., 2020; Buchwald et al., 2020). ICD is a type of cell death that promotes a T-cell mediated immune response against antigens derived from dying cells (Kroemer et al., 2013). Radiotherapy induces cell death which generates the release of damage-associated patterns (DAMPs), eventually resulting in ICD (Keam et al., 2020). There are three major DAMPs that contribute to ICD: calreticulin (CRT), high-mobility group box-1 (HMGB-1) and adenosine triphosphate (ATP). CRT can be found on the outer leaflet of dying tumor cells as an “eat me” signal for APCs, leading to presentation of TAAs to naïve T-cells which will result in an anti-tumor immune response (Gameiro et al., 2014). HMGB-1 is released from irradiated tumor cells into the immune environment where it stimulates DCs and macrophages to transcribe inflammatory genes (Wang et al., 2018). ATP is known as a “find me” signal for DCs and monocytes by binding to their purinergic P2X7 receptors, leading to the release of cytokines, such as IL-18 and IL-1β (Perregaux et al., 2000; Ghiringhelli et al., 2009; Aymeric et al., 2010). Radiotherapy can also induce anti-tumor immunity via the cGAS-STING pathway. Radiotherapy-induced DNA damage can be repaired by three central DDR kinases: DNA-dependent protein kinase (DNA-PK), ataxia telangiectasia-mutated (ATM) and ataxia telangiectasia & Rad3-related protein (ATR) (Zhang et al., 2022). These kinases potentiate two main repair mechanisms: non-homologous end-joining and homology-directed repair (Zhang et al., 2022). However, cancer cells often make mistakes during this repair process, which causes genomic instability and cell cycle checkpoint disruption eventually leading to the formation of DNA-containing micronuclei in the cytoplasm (Jeggo et al., 2016; Gekara, 2017). This DNA is then recognized by cyclic GMP-AMP synthase (cGAS) and is dimerized into a cGAS-DNA complex to catalyze the formation of cyclic guanosine monophosphate-adenosine monophosphate (cGAMP). cGAMP interacts with stimulator of IFN genes (STING) and activates it. STING then recruits tank-binding kinase-1 (TBK1) and IκB kinase (IKK) to phosphorylate interferon regulatory factor 3 (IRF3) and NF-κB inhibitor IκBα, respectively. Afterwards, IRF3 and NF-κB are translocated into the nucleus to induce the transcription of type I IFN genes and other inflammatory cytokines like IFN-β (Cai et al., 2014; McLaughlin et al., 2020; Xu et al., 2021). Type I IFN genes facilitate DC maturation, increase DC co-stimulatory molecule expression and enhance DC lymph node migration, which all induces CD8^+^ T-cell priming (Zitvogel et al., 2015; Sprooten et al., 2019).

Next to all the immune-activating effects of radiotherapy, it has also been shown to induce immunosuppression (Zhang et al., 2022). In this sense, radiotherapy can upregulate the expression of the immune checkpoint PD-L1, thereby inhibiting T-cell activation (Deng et al., 2014; Twyman-Saint Victor et al., 2015). Furthermore, radiotherapy may contribute to resistance mechanisms by reshaping immune cells in the tumor microenvironment towards an immunosuppressive phenotype (Dar et al., 2022). Whereas low-dose radiation can switch tumor-associated macrophages (TAMs) towards the M1 phenotype (pro-inflammatory/anti-tumorigenic), high-dose radiation promotes the M2 phenotype (anti-inflammatory/pro-tumorigenic) (Klug et al., 2013; Prakash et al., 2016; Seifert et al., 2016). Radiotherapy also induces an upregulation of a specific type of immunosuppressive cells called myeloid-derived suppressor cells (MDSCs) and Tregs in the tumor microenvironment (Xu et al., 2013; Muroyama et al., 2017). TGF-β and CCL2 are also upregulated after radiotherapy, resulting in Treg differentiation and attraction of MDSCs to the tumor microenvironment, respectively (Vanpouille-Box et al., 2015; Kalbasi et al., 2017; Dahmani and Delisle, 2018).

### 5.2 The influence of radio- and immunotherapy on each other

The abscopal effect of radiotherapy is not commonly observed but has been shown to be synergistically boosted by immunotherapy (Ngwa et al., 2018). Multiple preclinical and some clinical studies have shown an abscopal effect induced by dual radio- and immunotherapy (Demaria et al., 2005; Dewan et al., 2009; Yasuda et al., 2011; Deng et al., 2014; Grimaldi et al., 2014; Golden et al., 2015; Twyman-Saint Victor et al., 2015; Habets et al., 2016; Hao et al., 2016; Theurich et al., 2016; Young et al., 2016; Aboudaram et al., 2017; Dovedi et al., 2017; Koller et al., 2017; Rodriguez-Ruiz et al., 2017; Formenti et al., 2018; Rodríguez-Ruiz et al., 2018; Roger et al., 2018). However, there is a discrepancy in observed abscopal effects induced by dual radio- and immunotherapy between preclinical and clinical studies. It seems that preclinical studies show more promising results than clinical studies, which could be because murine models are not fully able to recapitulate metastatic cancer in patients. Mice are frequently injected with conventional cell lines, which lack genetic and environmental heterogeneity, and typically exhibit only minimal tumor growth (Olson et al., 2018; Arina et al., 2020). More recently, organoid mouse models of CRC have been established for testing radiotherapy (Kim et al., 2022; Nicolas et al., 2022b; Nicolas et al., 2022a). However, these models have yet to be utilized for immunotherapy testing and microbiome research. Radiotherapy and immunotherapy have been shown to enhance each other’s effects. Radiotherapy can enhance immunotherapy efficacy by reducing tumor burden, changing the tumor microenvironment and increasing T-cell infiltration into the tumor (Arina et al., 2020). Furthermore, most patients receiving immunotherapy need additional interventions to overcome primary or acquired resistance to immunotherapy (Vanpouille-Box et al., 2018). Radiotherapy can help to overcome at least some of the mechanisms by which cancer cells are or become resistant to immunotherapy. Radiotherapy generates T-cells specific for TAAs by inducing ICD. It overcomes T-cell exclusion from the tumor by promoting the release of chemokines that attract effector T-cells. It improves recognition and killing of cancer cells by CD8^+^ T-cells by promoting antigen presentation on MHC class I molecules, thereby upregulating death receptors and promoting the exposure of NK cell-activating ligands (Vanpouille-Box et al., 2018). On the other hand, immunotherapy positively influences radiotherapy by boosting radiotherapy-induced immune activation, blocking immunosuppressive effects of radiotherapy such as PD-L1 upregulation and eliminating microscopic tumors (Arina et al., 2020).

A couple of clinical trials investigating the effect of dual radio- and immunotherapy in CRC patients have been performed. These trials showed little success in shrinking non-irradiated tumors, but did show that the combination treatment was safe and induced an anti-tumor immune response (Monjazeb et al., 2021; Parikh et al., 2021; Segal et al., 2021). Currently, there are even more ongoing clinical trials investigating the effect of dual radio- and immunotherapy in CRC patients (NCT02437071, NCT02837263, NCT04575922, NCT03104439, NCT03101475, NCT02888743).

When MC38-tumor bearing mice were treated with irradiation (20 Gy) and anti-PD-L1 (four injections of 200 µg spread over 72 hours), the abscopal effect could be observed, leading to a significant decrease in tumor volume compared to monotherapy (Deng et al., 2014). In addition, dual radio- and immunotherapy activated CD8^+^ T-cells, which induced a reduction in MDSCs via TNF-mediated apoptosis, leading to more efficient tumor regression. Treatment of CT26-tumor bearing mice with radiotherapy (5 x 2 Gy) and anti-PD-1 or anti-PD-L1 (10 mg/kg, 3 injections/week for 3 weeks) was curative in 66% and 80% of mice, respectively (Dovedi et al., 2014). It was discovered that radiotherapy activates CD8^+^ T-cells, which produce IFN-γ, eventually leading to an upregulation of PD-L1 expression on tumor cells. Therefore, the efficacy of the combination treatment can be explained by the fact that immunotherapy blocks the radiotherapy-induced upregulation of PD-L1, allowing a better anti-tumor immune response. Furthermore, when long-term surviving mice (i.e., mice that completely rejected tumor after combination treatment) were rechallenged with tumor cells, they were able to reject the tumor again, indicating that the combination of radio- and immunotherapy generates protective immunologic memory. Another study by the same group investigating the effect of dual radio- and immunotherapy in mice with two tumors, of which only one was irradiated, showed that dual treatment was able to generate anti-tumor responses and tumor control in both irradiated and non-irradiated tumors (Dovedi et al., 2017). It was found that radiotherapy is able to induce polyclonal T-cell infiltration and expansion at the site of treatment, but not in the out-of-field tumor due to suppression through the PD-1/PD-L1 axis. Inhibition of this axis led to a polyclonal T-cell response capable of mediating out-of-field effects. When different radiation schemes (18 x 2 Gy, 3 x 8 Gy or 1 x 16.4 Gy) were applied on CT26-tumor bearing mice in combination with anti-PD-L1 treatment, the best tumor control and survival was observed for the 18 x 2 Gy scheme (Grapin et al., 2019). This indicates that hyperfractionation appears to be critical for lymphoid stimulation, while normo-fractionation seems to be deleterious to lymphoid cells, which are radiosensitive.

It is clear that preclinical studies investigating dual radio- and immunotherapy for CRC are hopeful, whereas clinical studies are not yet providing the same hopeful results. Furthermore, there is no information yet available about the influence of the gut microbiome for this combined treatment of CRC, making this an interesting topic to investigate further.

## 6 Modulating the gut microbiome to enhance therapeutic modalities for CRC

The gut microbiome can be altered in different ways (Rebersek, 2021). First, biotic interventions, such as diet (e.g., protein, fat, fiber intake) and vitamin intake can change its composition (Singh et al., 2017; Leeming et al., 2019; Pham et al., 2021). In addition, the gut microbiome can also be beneficially altered by pro-, pre- and postbiotics, which are microorganisms, substrates selectively utilized by host microorganisms, and preparations of inanimate microorganisms and/or their components, respectively (Hill et al., 2014; Gibson et al., 2017; Chen et al., 2021; Salminen et al., 2021). A subset of these pro- and prebiotics are designated as psychobiotics, which can boost the mental health status through modulation of the gut microbiome, and as such affect the gut-brain axis (Sarkar et al., 2016). A significant proportion of cancer patients suffer from mental health problems, such as anxiety and depression, which seems to have a negative influence on the gut microbiome (Clapp et al., 2017; Vucic et al., 2021). In the end, this can result in negative treatment outcomes, such as a higher incidence of side effects or less therapy response. Therefore, for these patients, psychobiotics can help to stimulate SCFA production in the gut, leading to the production of gut hormones that migrate to the central nervous system. This cascade induces the release of neurotransmitters, such as dopamine and serotonin, leading to a general decrease in inflammation and restoration of the gut barrier. Another way to modulate the microbiome is by using selective antibiotics that inhibit detrimental bacteria, increase the abundance of certain bacteria that promote cancer therapeutic efficacy or reduce treatment-related side effects by indirectly inhibiting detrimental bacterial metabolites (Wallace et al., 2010; Bullman et al., 2017; Zitvogel et al., 2017). Lastly, fecal microbiota transplantation (FMT), which is the administration of fecal material from a donor into the intestinal tract of a recipient to alter the host’s gut microbiome composition, can be used for therapeutic benefit (Wang et al., 2019). There are multiple methods to deliver FMT, yet the most optimal delivery route remains unclear (Ramai et al., 2019). FMT can for instance be delivered via the upper (via esophagogastroduodenoscopy (EGD), nasogastric, nasojejunal, nasoduodenal tubes or oral capsules) or lower (colonoscopy, sigmoidoscopy and enema) GI route (Gulati et al., 2020). Furthermore, FMT can either be autologous (a-FMT), defined as transplantation of one’s own fecal material taken during healthy state, or heterologous (h-FMT), defined as transplantation of fecal material from a healthy donor to a diseased host (Basson et al., 2020). FMT has been shown to be an effective treatment for recurrent *Clostridium difficile* infection, which led to its approval by the FDA for this disease (Kelly et al., 2014; Agrawal et al., 2016). Currently, this is the only indication to use FMT in patients. However, the potential beneficial role of FMT for other diseases, such as IBD, functional bowel disorders, metabolic syndrome, autism and multiple sclerosis, is under investigation in clinical trials (Cui et al., 2015; Tian et al., 2016; Paramsothy et al., 2017; Li et al., 2021; Al et al., 2022). Furthermore, FMT has been shown to a have a direct beneficial effect on CRC by reducing inflammation and reducing the abundance of cancer-promoting bacteria (Chen et al., 2019; Kaźmierczak-Siedlecka et al., 2020). Given the importance of FMT, its role in radio- and immunotherapy for CRC is discussed in more detail hereunder.

## 7 FMT to counteract radiotherapy-related side effects

Radiation induces dysbiosis and GI side effects, which might be reverted by FMT. The use of FMT to reduce dysbiosis and GI side effects has therefore been investigated. A clinical trial investigating the use of FMT as a treatment for chronic RE after abdominal/pelvic irradiation has been performed in five female patients with gynecological cancer (Ding et al., 2020). FMT from healthy donors (18-24 years) was administered for a maximum of three times over a maximum of two weeks through nasojejunal transendoscopic enteral tubing (TET). FMT led to amelioration in rectal hemorrhage, fecal incontinence, diarrhea and abdominal and rectal pain in three out of five recruited patients. However, the efficacy of FMT was not long lasting, indicating that patients may require repeated cycles of FMT. Another case report of a 59-year-old woman with chronic RE after pelvic irradiation showed that FMT from her 18-year-old son improved her chronic RE-related symptoms (Liu et al., 2022). For radiation proctitis after pelvic irradiation, a case report of a 45-year-old woman has shown that four courses of FMT from healthy donors (21 – 24 years) ameliorated the patient’s hematochezia, abdominal pain and diarrhea (Zheng et al., 2020). Furthermore, FMT decreased the abundance of the *Firmicutes* phylum and increased the abundance of the *Bacteroidetes* phylum. In the previously mentioned studies, FMT was able to increase the diversity of the patient’s microbiome and shift its composition to one similar of that of the donor (Ding et al., 2020; Zheng et al., 2020).

In another study, mice received fresh FMT from healthy, age-matched mice every day for 10 days via oral gavage after radiation treatment (Cui et al., 2017). They discovered that FMT increased the survival rate and body weight, improved GI tract function and epithelial integrity and enhanced angiogenesis without accelerating tumor growth. FMT was also able to restore the gut microbiome, since it increased the abundance of *Bacteroides*, *Lactobacillus* and *Prevotella*, which were decreased after irradiation. Differences between male and female mice could also be observed, in that male mice that received FMT had increased expression of genes involved in the innate and adaptive immune system, whereas female mice showed increased expression of genes involved in metabolism. In follow-up studies, it was discovered that the abundance of the metabolite indole 3-propionic acid (IPA) and the SCFA valeric acid increased after FMT treatment of irradiated mice (Li et al., 2020; Xiao et al., 2020). Both metabolites seemed to be able to increase survival and reduce GI side effects of irradiated mice.

We can conclude that FMT appears to have a beneficial influence on radiotherapy-induced toxicity in both mice and humans. FMT seems to restore the gut microbiome after irradiation, but more research concerning microbial changes after FMT is needed to get better insights in potential beneficial microbes. Moreover, no studies have reported the involvement of FMT in enhancing radiotherapy efficacy, presenting an intriguing area for additional research.

## 8 FMT to increase immunotherapeutic efficacy

Since the efficacy and toxicity of ICBs is dependent on the composition of the gut microbiome, both may be improved by altering the gut microbiome using FMT (Park et al., 2020). However, currently, there is only some evidence on the beneficial effect of FMT on immunotherapy efficacy for CRC.

A recent clinical trial investigated the combination of FMT and anti-PD-1 (NCT04130763) (Peng et al., 2023). In this trial, patients received FMT capsules from healthy donors with a microbiome similar to that of anti-PD-1 responders. FMT was first administered on its own for one week (for three consecutive days), after which anti-PD-1 was added to the FMT treatment for 6 cycles. It was discovered that FMT was well tolerated in patients and enhanced anti-PD-1 efficacy. Furthermore, FMT seemed to increase alpha diversity and IFN-γ^+^ CD8^+^ T-cells.

Treatment of CT26-tumor bearing mice with anti-PD-1 treatment (4 x 200 µg) and FMT (4 x (5x10^9^ CFU)) from healthy human donors led to improved survival and reduced tumor growth (Huang et al., 2022). These mice had an increased abundance of *Parabacteroides distasonis* and a reduced abundance of *Clostridium* sp. HGF2*, Enterococcus hirae, Dorea* sp. 52 and *Lactobacillus murinus.* In addition, these mice showed an increased abundance of certain *Bacteroides* spp. (*B. thetaiotaomicron, B. fragilis, B. cellulosilyticus, B. salyersiae, B. stercoris, B. uniformis, B. massiliensis)*, but a reduced abundance of *Bacteroides ovatus*. Some of these bacteria seem to inhibit tumor growth. For instance, *B. thetaiotaomicron* induces DCs, maintains intestinal homeostasis by mediating microbe-host crosstalk and inhibits CRC carcinogenesis via its metabolite propionate (Durant et al., 2020; Ryu et al., 2022). *B. fragilis* induces Tregs to secrete IL-10 through its molecule polysaccharide A (PSA) and activates NKT cells through the production of alpha-galactose ceramides (Dasgupta et al., 2014; Oh et al., 2021). *B. cellulosilyticus* can activate Tregs to secrete IL-10 via its zwitterionic capsular polysaccharide (Neff et al., 2016). On the other hand, some bacteria can promote tumor growth. *B. ovatus* can influence immunity by producing IgA and has already been associated with shorter progression-free survival in melanoma patients receiving immunotherapy (Peters et al., 2019; Yang et al., 2020). *L. murinus* impairs gut metabolic function, thereby contributing to intestinal dysbiosis (Hayashi et al., 2017). Based on microbiome composition, it was also shown that mice treated with anti-PD-1 and FMT showed enriched expression of nucleotides and amino acid biosynthesis pathways and reduced expression of methionine and S-adenosyl-L-methionine (SAM) pathways (Huang et al., 2022). Methionine and SAM are involved in cancer pathogenesis and cancer metastasis/recurrence, respectively (Gao et al., 2019; Sanderson et al., 2019; Zhang et al., 2021). Mice treated with anti-PD-1 and FMT also showed higher amounts of aspirin, which inhibits the growth of CRC-associated bacterium *F. nucleatum*, and punicic acid, which elicits anti-tumor effects (Mete et al., 2019; Brennan et al., 2021; Yuan et al., 2021; Huang et al., 2022). In addition, these mice showed reduced amounts of glycine and serine, which can inhibit tumor growth in CRC mouse models (Maddocks et al., 2017; Muthusamy et al., 2020). Finally, kynurenic acid is reduced in these mice, which seems contradicting since this inhibits CRC (Walczak et al., 2014). FMT could also be a potential intervention to reduce immunotherapy-related adverse events. One of the most common ICB-related side effects is colitis. For melanoma, presence of the *Bacteroidetes* phylum in the gut has been associated with resistance to ICB-induced colitis, whereas the opposite was observed for the presence of the *Firmicutes* phylum (Dubin et al., 2016; Chaput et al., 2017). Pathways involved in polyamine transport and vitamin B biosynthesis are associated with an increased risk of colitis (Dubin et al., 2016). *Bifidobacterium* (*B. bifidum, B. longum*, *B. lactis* and *B. breve*) was able to reduce ICB-induced toxicity in a preclinical colitis model (Wang et al., 2018). Wang et al. were the first to discover that FMT was able to treat ICB-associated colitis (Wang et al., 2018). The investigators administered FMT (50 grams) to two patients (patient 1: one treatment; patient 2: two treatments) via colonoscopy and found that FMT reconstitutes the gut microbiome and induces the proportion of Tregs in the colonic mucosa. There are currently some ongoing clinical trials to investigate the ability of FMT to reduce immunotherapy-related side effect [NCT04163289 (renal cell carcinoma (RCC)], NCT03819296 [melanoma/genitourinary cancer)]. However, no clinical trials involving CRC are currently being conducted.

It is clear that mouse as well as human studies that investigate the beneficial role of FMT for ICB efficacy and side effects are lacking. In humans, FMT seems to enhance antitumor responses and ICB efficacy. In mice, FMT seems to increase the abundance of *Parabacteroides*, which improves ICB efficacy. Strikingly, FMT increases the abundance of *Bacteroides*, which is associated with worse ICB efficacy. Nevertheless, it is clear that more information from mouse and human studies is needed to advance our knowledge on the impact of FMT on ICB efficacy and side effects.

## 9 Conclusion and future perspectives

Overall, the gut microbiome has a significant impact on radio- and immunotherapy treatment of CRC. Bacteria involved in radiotoxicity have been identified, but microbiome data from mice and patients often contrasts, which can partly be explained by their intrinsic microbiome differences. On the other hand, most radiotherapy research has only been performed on healthy mice, thereby overlooking the dysbiotic microbiome composition that is associated with CRC. Therefore, further research exploring the microbiome’s role in radiotherapy toxicity should be conducted on cancer-bearing mice in order to facilitate a more robust comparison between humans and mice. Furthermore, since CRC patients as well as mice exhibit dysbiosis prior to radio- or immunotherapy that may affect their response to these treatments, more studies investigating the microbiome composition pre- and post-treatment are warranted to identify potential predictive biomarkers for treatment response. It has also become evident that there is a lack of data on how the microbiome influences radiotherapy efficacy, immunotherapy toxicity and combined radio-immunotherapy outcomes. As such, further investigation in mice and patients will be necessary to identify if and which bacteria are involved in these processes. Nevertheless, the administration of microbiota from a healthy donor to a CRC recipient via FMT has shown to restore the gut microbiome, thereby reducing radiation-induced toxicity and enhancing immunotherapy efficacy in mice and patients. Furthermore, FMT was able to reduce immunotherapy-induced colitis in preclinical and clinical settings for cancer types other than CRC. More research in mice and patients will be needed to confirm these findings for CRC. To conclude, although the available data corroborates that the gut microbiome and FMT influence radio- and immunotherapy toxicities and efficacies for CRC, the available information is limited and often contrasting, warranting more studies on CRC mice and patients in order to progress our current understanding.

## Author contributions

LVD: Conceptualization, Data curation, Visualization, Writing – original draft, Writing – review & editing. CS: Writing – review & editing. SW: Writing – review & editing. MM: Writing – review & editing. NL: Writing – review & editing. SK-S: Supervision, Writing – review & editing. SM-K: Supervision, Writing – review & editing. RVH: Supervision, Visualization, Writing – review & editing.

## References

[B1] Aboudaram A. Modesto A. Chaltiel L. Gomez-Roca C. Boulinguez S. Sibaud V.. (2017). Concurrent radiotherapy for patients with metastatic melanoma and receiving anti-programmed-death 1 therapy: a safe and effective combination. Melanoma Res. 27 (5), 485–491. doi: 10.1097/CMR.0000000000000386 28858075

[B2] Agrawal M. Aroniadis O. C. Brandt L. J. Kelly C. Freeman S. Surawicz C.. (2016). The long-term efficacy and safety of fecal microbiota transplant for recurrent, severe, and complicated *clostridium* difficile infection in 146 elderly individuals. J. Clin. Gastroenterol. 50 (5), 403–407. doi: 10.1097/MCG.0000000000000410 26352106

[B3] Al K. F. Craven L. J. Gibbons S. Parvathy S. N. Wing A. C. Graf C.. (2022). Fecal microbiota transplantation is safe and tolerable in patients with multiple sclerosis: A pilot randomized controlled trial. Mult Scler J. Exp. Transl. Clin. 8 (2), 20552173221086662. doi: 10.1177/20552173221086662 35571974 PMC9102167

[B4] Andreassen C. N. Alsner J. (2009). Genetic variants and normal tissue toxicity after radiotherapy: a systematic review. Radiother Oncol. 92 (3), 299–309. doi: 10.1016/j.radonc.2009.06.015 19683821

[B5] Arina A. Gutiontov S. I. Weichselbaum R. R. (2020). Radiotherapy and immunotherapy for cancer: from “Systemic” to “Multisite”. Clin. Cancer Res. 26 (12), 2777–2782. doi: 10.1158/1078-0432.CCR-19-2034 32047000 PMC10759929

[B6] Artemev A. Naik S. Pougno A. Honnavar P. Shanbhag N. M. (2022). The association of microbiome dysbiosis with colorectal cancer. Cureus 14 (2), e22156. doi: 10.7759/cureus.22156 35174040 PMC8840808

[B7] Awedew A. F. Asefa Z. Belay W. B. (2022). Burden and trend of colorectal cancer in 54 countries of Africa 2010-2019: a systematic examination for Global Burden of Disease. BMC Gastroenterol. 22 (1), 204. doi: 10.1186/s12876-022-02275-0 35468750 PMC9036749

[B8] Aymeric L. Apetoh L. Ghiringhelli F. Tesniere A. Martins I. Kroemer G.. (2010). Tumor cell death and ATP release prime dendritic cells and efficient anticancer immunity. Cancer Res. 70 (3), 855–858. doi: 10.1158/0008-5472.CAN-09-3566 20086177

[B9] Baba K. Nomura M. Ohashi S. Hiratsuka T. Nakai Y. Saito T.. (2020). Experimental model for the irradiation-mediated abscopal effect and factors influencing this effect. Am. J. Cancer Res. 10 (2), 440–453. 32195019 PMC7061743

[B10] Bachem A. Makhlouf C. Binger K. J. de Souza D. P. Tull D. Hochheiser K.. (2019). Microbiota-derived short-chain fatty acids promote the memory potential of antigen-activated CD8(+) T cells. Immunity. 51 (2), 285–97.e5. doi: 10.1016/j.immuni.2019.06.002 31272808

[B11] Baek M. K. Park J. S. Park J. H. Kim M. H. Kim H. D. Bae W. K.. (2010). Lithocholic acid upregulates uPAR and cell invasiveness via MAPK and AP-1 signaling in colon cancer cells. Cancer Lett. 290 (1), 123–128. doi: 10.1016/j.canlet.2009.08.030 19782465

[B12] Baruch E. N. Wang J. Wargo J. A. (2021). Gut microbiota and antitumor immunity: potential mechanisms for clinical effect. Cancer Immunol. Res. 9 (4), 365–370. doi: 10.1158/2326-6066.CIR-20-0877 34003768

[B13] Basson A. R. Zhou Y. Seo B. Rodriguez-Palacios A. Cominelli F. (2020). Autologous fecal microbiota transplantation for the treatment of inflammatory bowel disease. Transl. Res. 226, 1–11. doi: 10.1016/j.trsl.2020.05.008 32585148 PMC7308243

[B14] Bentzen S. M. Overgaard J. (1994). Patient-to-patient variability in the expression of radiation-induced normal tissue injury. Semin. Radiat. Oncol. 4 (2), 68–80. doi: 10.1016/S1053-4296(05)80034-7 10717093

[B15] Berg G. Rybakova D. Fischer D. Cernava T. Vergès M. C. Charles T.. (2020). Microbiome definition re-visited: old concepts and new challenges. Microbiome. 8 (1), 103. doi: 10.1186/s40168-020-00875-0 32605663 PMC7329523

[B16] Berg R. D. (1999). Bacterial translocation from the gastrointestinal tract. Adv. Exp. Med. Biol. 473, 11–30. doi: 10.1007/978-1-4615-4143-1_2 10659341

[B17] Bhat M. I. Kapila R. (2017). Dietary metabolites derived from gut microbiota: critical modulators of epigenetic changes in mammals. Nutr. Rev. 75 (5), 374–389. doi: 10.1093/nutrit/nux001 28444216

[B18] Boland C. R. Thibodeau S. N. Hamilton S. R. Sidransky D. Eshleman J. R. Burt R. W.. (1998). A National Cancer Institute Workshop on Microsatellite Instability for cancer detection and familial predisposition: development of international criteria for the determination of microsatellite instability in colorectal cancer. Cancer Res. 58 (22), 5248–5257. 9823339

[B19] Bonneville R. Krook M. A. Chen H. Z. Smith A. Samorodnitsky E. Wing M. R.. (2020). Detection of microsatellite instability biomarkers via next-generation sequencing. Methods Mol. Biol. 2055, 119–132. doi: 10.1007/978-1-4939-9773-2_5 31502149 PMC7010320

[B20] Bosetti C. Rosato V. Gallus S. Cuzick J. La Vecchia C. (2012). Aspirin and cancer risk: a quantitative review to 2011. Ann. Oncol. 23 (6), 1403–1415. doi: 10.1093/annonc/mds113 22517822

[B21] Boyle T. Keegel T. Bull F. Heyworth J. Fritschi L. (2012). Physical activity and risks of proximal and distal colon cancers: a systematic review and meta-analysis. J. Natl. Cancer Inst. 104 (20), 1548–1561. doi: 10.1093/jnci/djs354 22914790

[B22] Brennan C. A. Nakatsu G. Gallini Comeau C. A. Drew D. A. Glickman J. N. Schoen R. E.. (2021). Aspirin modulation of the colorectal cancer-associated microbe fusobacterium nucleatum. mBio 12 (2), e00547-21. doi: 10.1128/mBio.00547-21 33824205 PMC8092249

[B23] Brenner H. Chang-Claude J. Seiler C. M. Rickert A. Hoffmeister M. (2011). Protection from colorectal cancer after colonoscopy: a population-based, case-control study. Ann. Intern. Med. 154 (1), 22–30. doi: 10.7326/0003-4819-154-1-201101040-00004 21200035

[B24] Brenner H. Kloor M. Pox C. P. (2014). Colorectal cancer. Lancet 383 (9927), 1490–1502. doi: 10.1016/S0140-6736(13)61649-9 24225001

[B25] Brix N. Tiefenthaller A. Anders H. Belka C. Lauber K. (2017). Abscopal, immunological effects of radiotherapy: Narrowing the gap between clinical and preclinical experiences. Immunol. Rev. 280 (1), 249–279. doi: 10.1111/imr.12573 29027221

[B26] Buchwald Z. S. Nasti T. H. Lee J. Eberhardt C. S. Wieland A. Im S. J.. (2020). Tumor-draining lymph node is important for a robust abscopal effect stimulated by radiotherapy. J. Immunother. Cancer 8 (2), e000867. doi: 10.1136/jitc-2020-000867 33028691 PMC7542667

[B27] Bullman S. Pedamallu C. S. Sicinska E. Clancy T. E. Zhang X. Cai D.. (2017). Analysis of Fusobacterium persistence and antibiotic response in colorectal cancer. Science 358 (6369), 1443–1448. doi: 10.1126/science.aal5240 29170280 PMC5823247

[B28] Cai X. Chiu Y. H. Chen Z. J. (2014). The cGAS-cGAMP-STING pathway of cytosolic DNA sensing and signaling. Mol. Cell. 54 (2), 289–296. doi: 10.1016/j.molcel.2014.03.040 24766893

[B29] Casero R. A. Jr. Marton L. J. (2007). Targeting polyamine metabolism and function in cancer and other hyperproliferative diseases. Nat. Rev. Drug Discov. 6 (5), 373–390. doi: 10.1038/nrd2243 17464296

[B30] Center M. M. Jemal A. Smith R. A. Ward E. (2009). Worldwide variations in colorectal cancer. CA Cancer J. Clin. 59 (6), 366–378. doi: 10.3322/caac.20038 19897840

[B31] Chan D. S. Lau R. Aune D. Vieira R. Greenwood D. C. Kampman E.. (2011). Red and processed meat and colorectal cancer incidence: meta-analysis of prospective studies. PloS One 6 (6), e20456. doi: 10.1371/journal.pone.0020456 21674008 PMC3108955

[B32] Chan S. Rowbottom L. McDonald R. Bjarnason G. A. Tsao M. Danjoux C.. (2017). Does the time of radiotherapy affect treatment outcomes? A review of the literature. Clin. Oncol. (R Coll. Radiol). 29 (4), 231–238. doi: 10.1016/j.clon.2016.12.005 28034487

[B33] Chaput N. Lepage P. Coutzac C. Soularue E. Le Roux K. Monot C.. (2017). Baseline gut microbiota predicts clinical response and colitis in metastatic melanoma patients treated with ipilimumab. Ann. Oncol. 28 (6), 1368–1379. doi: 10.1093/annonc/mdx108 28368458

[B34] Chen D. Wu J. Jin D. Wang B. Cao H. (2019). Fecal microbiota transplantation in cancer management: Current status and perspectives. Int. J. Cancer. 145 (8), 2021–2031. doi: 10.1002/ijc.32003 30458058 PMC6767494

[B35] Chen J. Chen X. Ho C. L. (2021). Recent development of probiotic bifidobacteria for treating human diseases. Front. Bioeng Biotechnol. 9, 770248. doi: 10.3389/fbioe.2021.770248 35004640 PMC8727868

[B36] Cheng W. T. Kantilal H. K. Davamani F. (2020). The mechanism of bacteroides fragilis toxin contributes to colon cancer formation. Malays J. Med. Sci. 27 (4), 9–21. doi: 10.21315/mjms2020.27.4.2 32863742 PMC7444842

[B37] Cheng Y. Ling Z. Li L. (2020). The intestinal microbiota and colorectal cancer. Front. Immunol. 11, 615056. doi: 10.3389/fimmu.2020.615056 33329610 PMC7734048

[B38] Chew S. S. Tan L. T. Law J. W. Pusparajah P. Goh B. H. Ab Mutalib N. S.. (2020). Targeting gut microbial biofilms-A key to hinder colon carcinogenesis? Cancers (Basel) 12 (8), 2272. doi: 10.3390/cancers12082272 32823729 PMC7465663

[B39] Chhabra N. Kennedy J. (2021). A review of cancer immunotherapy toxicity: immune checkpoint inhibitors. J. Med. Toxicol. 17 (4), 411–424. doi: 10.1007/s13181-021-00833-8 33826117 PMC8455777

[B40] Clapp M. Aurora N. Herrera L. Bhatia M. Wilen E. Wakefield S. (2017). Gut microbiota’s effect on mental health: The gut-brain axis. Clin. Pract. 7 (4), 987. doi: 10.4081/cp.2017.987 29071061 PMC5641835

[B41] Clooney A. G. Eckenberger J. Laserna-Mendieta E. Sexton K. A. Bernstein M. T. Vagianos K.. (2021). Ranking microbiome variance in inflammatory bowel disease: a large longitudinal intercontinental study. Gut. 70 (3), 499–510. doi: 10.1136/gutjnl-2020-321106 32536605 PMC7873428

[B42] Crawford P. A. Gordon J. I. (2005). Microbial regulation of intestinal radiosensitivity. Proc. Natl. Acad. Sci. U. S. A. 102 (37), 13254–13259. doi: 10.1073/pnas.0504830102 16129828 PMC1193536

[B43] Cresci G. A. Bawden E. (2015). Gut microbiome: what we do and don’t know. Nutr. Clin. Pract. 30 (6), 734–746. doi: 10.1177/0884533615609899 26449893 PMC4838018

[B44] Cui B. Li P. Xu L. Zhao Y. Wang H. Peng Z.. (2015). Step-up fecal microbiota transplantation strategy: a pilot study for steroid-dependent ulcerative colitis. J. Transl. Med. 13, 298. doi: 10.1186/s12967-015-0646-2 26363929 PMC4567790

[B45] Cui M. Xiao H. Li Y. Zhou L. Zhao S. Luo D.. (2017). Faecal microbiota transplantation protects against radiation-induced toxicity. EMBO Mol. Med. 9 (4), 448–461. doi: 10.15252/emmm.201606932 28242755 PMC5376756

[B46] Cui M. Xiao H. Luo D. Zhang X. Zhao S. Zheng Q.. (2016). Circadian rhythm shapes the gut microbiota affecting host radiosensitivity. Int. J. Mol. Sci. 17 (11):1786. doi: 10.3390/ijms17111786 27792172 PMC5133787

[B47] Cuzzolin L. Zambreri D. Donini M. Griso C. Benoni G. (1992). Influence of radiotherapy on intestinal microflora in cancer patients. J. Chemother. 4 (3), 176–179. doi: 10.1080/1120009X.1992.11739160 1517812

[B48] Dahmani A. Delisle J. S. (2018). TGF-β in T cell biology: implications for cancer immunotherapy. Cancers (Basel) 10 (6), 194. doi: 10.3390/cancers10060194 29891791 PMC6025055

[B49] Dar T. B. Biteghe F. A. N. Kakar-Bhanot R. Aniogo E. C. Malindi Z. Akinrinmade O. A.. (2022). Synergistic effects of radiotherapy and targeted immunotherapy in improving tumor treatment efficacy: a review. Clin. Transl. Oncol. 24 (12), 2255–2271. doi: 10.1007/s12094-022-02888-7 35913663

[B50] Dasgupta S. Erturk-Hasdemir D. Ochoa-Reparaz J. Reinecker H. C. Kasper D. L. (2014). Plasmacytoid dendritic cells mediate anti-inflammatory responses to a gut commensal molecule via both innate and adaptive mechanisms. Cell Host Microbe 15 (4), 413–423. doi: 10.1016/j.chom.2014.03.006 24721570 PMC4020153

[B51] de Almeida C. V. Taddei A. Amedei A. (2018). The controversial role of Enterococcus faecalis in colorectal cancer. Therap Adv. Gastroenterol. 11, 1756284818783606. doi: 10.1177/1756284818783606 PMC6044108 30013618

[B52] De’ Angelis G. L. Bottarelli L. Azzoni C. De’ Angelis N. Leandro G. Di Mario F.. (2018). Microsatellite instability in colorectal cancer. Acta BioMed. 89 (9-s), 97–101. doi: 10.23750/abm.v89i9-S.7960 30561401 PMC6502181

[B53] DeGruttola A. K. Low D. Mizoguchi A. Mizoguchi E. (2016). Current understanding of dysbiosis in disease in human and animal models. Inflammation Bowel Dis. 22 (5), 1137–1150. doi: 10.1097/MIB.0000000000000750 PMC4838534 27070911

[B54] Dejea C. M. Wick E. C. Hechenbleikner E. M. White J. R. Mark Welch J. L. Rossetti B. J.. (2014). Microbiota organization is a distinct feature of proximal colorectal cancers. Proc. Natl. Acad. Sci. U. S. A. 111 (51), 18321–18326. doi: 10.1073/pnas.1406199111 25489084 PMC4280621

[B55] Demaria S. Kawashima N. Yang A. M. Devitt M. L. Babb J. S. Allison J. P.. (2005). Immune-mediated inhibition of metastases after treatment with local radiation and CTLA-4 blockade in a mouse model of breast cancer. Clin. Cancer Res. 11 (2 Pt 1), 728–734. doi: 10.1158/1078-0432.728.11.2 15701862

[B56] Deng L. Liang H. Burnette B. Beckett M. Darga T. Weichselbaum R. R.. (2014). Irradiation and anti-PD-L1 treatment synergistically promote antitumor immunity in mice. J. Clin. Invest. 124 (2), 687–695. doi: 10.1172/JCI67313 24382348 PMC3904601

[B57] Deng Q. Wang C. Yu K. Wang Y. Yang Q. Zhang J.. (2020). Streptococcus bovis Contributes to the Development of Colorectal Cancer via Recruiting CD11b(+)TLR-4(+) Cells. Med. Sci. Monit. 26, e921886. doi: 10.12659/MSM.921886 32737964 PMC7418781

[B58] Deschasaux M. Bouter K. E. Prodan A. Levin E. Groen A. K. Herrema H.. (2018). Depicting the composition of gut microbiota in a population with varied ethnic origins but shared geography. Nat. Med. 24 (10), 1526–1531. doi: 10.1038/s41591-018-0160-1 30150717

[B59] Devlin A. S. Fischbach M. A. (2015). A biosynthetic pathway for a prominent class of microbiota-derived bile acids. Nat. Chem. Biol. 11 (9), 685–690. doi: 10.1038/nchembio.1864 26192599 PMC4543561

[B60] Dewan M. Z. Galloway A. E. Kawashima N. Dewyngaert J. K. Babb J. S. Formenti S. C.. (2009). Fractionated but not single-dose radiotherapy induces an immune-mediated abscopal effect when combined with anti-CTLA-4 antibody. Clin. Cancer Res. 15 (17), 5379–5388. doi: 10.1158/1078-0432.CCR-09-0265 19706802 PMC2746048

[B61] Diaz L. A. Jr. Shiu K. K. Kim T. W. Jensen B. V. Jensen L. H. Punt C.. (2022). Pembrolizumab versus chemotherapy for microsatellite instability-high or mismatch repair-deficient metastatic colorectal cancer (KEYNOTE-177): final analysis of a randomised, open-label, phase 3 study. Lancet Oncol. 23 (5), 659–670. doi: 10.1016/S1470-2045(22)00197-8 35427471 PMC9533375

[B62] Ding X. Li Q. Li P. Chen X. Xiang L. Bi L.. (2020). Fecal microbiota transplantation: A promising treatment for radiation enteritis? Radiother Oncol. 143, 12–18. doi: 10.3389/fimmu.2020.01407 32044171

[B63] Ding S. Xu S. Fang J. Jiang H. (2020). The protective effect of polyphenols for colorectal cancer. Front. Immunol. 11, 1407. doi: 10.3389/fimmu.2020.01407 32754151 PMC7366338

[B64] Dovedi S. J. Adlard A. L. Lipowska-Bhalla G. McKenna C. Jones S. Cheadle E. J.. (2014). Acquired resistance to fractionated radiotherapy can be overcome by concurrent PD-L1 blockade. Cancer Res. 74 (19), 5458–5468. doi: 10.1158/0008-5472.CAN-14-1258 25274032

[B65] Dovedi S. J. Cheadle E. J. Popple A. L. Poon E. Morrow M. Stewart R.. (2017). Fractionated radiation therapy stimulates antitumor immunity mediated by both resident and infiltrating polyclonal T-cell populations when combined with PD-1 blockade. Clin. Cancer Res. 23 (18), 5514–5526. doi: 10.1158/1078-0432.CCR-16-1673 28533222

[B66] Dubin K. Callahan M. K. Ren B. Khanin R. Viale A. Ling L.. (2016). Intestinal microbiome analyses identify melanoma patients at risk for checkpoint-blockade-induced colitis. Nat. Commun. 7, 10391. doi: 10.1038/ncomms10391 26837003 PMC4740747

[B67] Durant L. Stentz R. Noble A. Brooks J. Gicheva N. Reddi D.. (2020). Bacteroides thetaiotaomicron-derived outer membrane vesicles promote regulatory dendritic cell responses in health but not in inflammatory bowel disease. Microbiome. 8 (1), 88. doi: 10.1186/s40168-020-00868-z 32513301 PMC7282036

[B68] Fedirko V. Tramacere I. Bagnardi V. Rota M. Scotti L. Islami F.. (2011). Alcohol drinking and colorectal cancer risk: an overall and dose-response meta-analysis of published studies. Ann. Oncol. 22 (9), 1958–1972. doi: 10.1093/annonc/mdq653 21307158

[B69] Feng Q. Liang S. Jia H. Stadlmayr A. Tang L. Lan Z.. (2015). Gut microbiome development along the colorectal adenoma-carcinoma sequence. Nat. Commun. 6, 6528. doi: 10.1038/ncomms7528 25758642

[B70] Formenti S. C. Lee P. Adams S. Goldberg J. D. Li X. Xie M. W.. (2018). Focal irradiation and systemic TGFβ Blockade in metastatic breast cancer. Clin. Cancer Res. 24 (11), 2493–2504. doi: 10.1158/1078-0432.CCR-17-3322 29476019 PMC5999326

[B71] Frey B. Rückert M. Deloch L. Rühle P. F. Derer A. Fietkau R.. (2017). Immunomodulation by ionizing radiation-impact for design of radio-immunotherapies and for treatment of inflammatory diseases. Immunol. Rev. 280 (1), 231–248. doi: 10.1111/imr.12572 29027224

[B72] Galon J. Costes A. Sanchez-Cabo F. Kirilovsky A. Mlecnik B. Lagorce-Pagès C.. (2006). Type, density, and location of immune cells within human colorectal tumors predict clinical outcome. Science 313 (5795), 1960–1964. doi: 10.1126/science.1129139 17008531

[B73] Galon J. Fridman W. H. Pagès F. (2007). The adaptive immunologic microenvironment in colorectal cancer: a novel perspective. Cancer Res. 67 (5), 1883–1886. doi: 10.1158/0008-5472.CAN-06-4806 17332313

[B74] Gameiro S. R. Jammeh M. L. Wattenberg M. M. Tsang K. Y. Ferrone S. Hodge J. W. (2014). Radiation-induced immunogenic modulation of tumor enhances antigen processing and calreticulin exposure, resulting in enhanced T-cell killing. Oncotarget 5 (2), 403–416. doi: 10.18632/oncotarget.1719 24480782 PMC3964216

[B75] Ganesh K. Stadler Z. K. Cercek A. Mendelsohn R. B. Shia J. Segal N. H.. (2019). Immunotherapy in colorectal cancer: rationale, challenges and potential. Nat. Rev. Gastroenterol. Hepatol. 16 (6), 361–375. doi: 10.1038/s41575-019-0126-x 30886395 PMC7295073

[B76] Gao X. Sanderson S. M. Dai Z. Reid M. A. Cooper D. E. Lu M.. (2019). Dietary methionine influences therapy in mouse cancer models and alters human metabolism. Nature 572 (7769), 397–401. 31367041 10.1038/s41586-019-1437-3 PMC6951023

[B77] García-Peris P. Velasco C. Lozano M. A. Moreno Y. Paron L. de la Cuerda C.. (2012). Effect of a mixture of inulin and fructo-oligosaccharide on Lactobacillus and *Bifidobacterium* intestinal microbiota of patients receiving radiotherapy: a randomised, double-blind, placebo-controlled trial. Nutr. Hosp. 27 (6), 1908–1915. doi: 10.3305/nh.2012.27.6.5992 23588438

[B78] Gekara N. O. (2017). DNA damage-induced immune response: Micronuclei provide key platform. J. Cell Biol. 216 (10), 2999–3001. doi: 10.1083/jcb.201708069 28860276 PMC5626557

[B79] Gerassy-Vainberg S. Blatt A. Danin-Poleg Y. Gershovich K. Sabo E. Nevelsky A.. (2018). Radiation induces proinflammatory dysbiosis: transmission of inflammatory susceptibility by host cytokine induction. Gut. 67 (1), 97–107. doi: 10.1136/gutjnl-2017-313789 28438965

[B80] Ghiringhelli F. Apetoh L. Tesniere A. Aymeric L. Ma Y. Ortiz C.. (2009). Activation of the NLRP3 inflammasome in dendritic cells induces IL-1beta-dependent adaptive immunity against tumors. Nat. Med. 15 (10), 1170–1178. doi: 10.1038/nm.2028 19767732

[B81] Gibson G. R. Hutkins R. Sanders M. E. Prescott S. L. Reimer R. A. Salminen S. J.. (2017). Expert consensus document: The International Scientific Association for Probiotics and Prebiotics (ISAPP) consensus statement on the definition and scope of prebiotics. Nat. Rev. Gastroenterol. Hepatol. 14 (8), 491–502. doi: 10.1038/nrgastro.2017.75 28611480

[B82] Golden E. B. Apetoh L. (2015). Radiotherapy and immunogenic cell death. Semin. Radiat. Oncol. 25 (1), 11–17. doi: 10.1016/j.semradonc.2014.07.005 25481261

[B83] Golden E. B. Chhabra A. Chachoua A. Adams S. Donach M. Fenton-Kerimian M.. (2015). Local radiotherapy and granulocyte-macrophage colony-stimulating factor to generate abscopal responses in patients with metastatic solid tumours: a proof-of-principle trial. Lancet Oncol. 16 (7), 795–803. doi: 10.1016/S1470-2045(15)00054-6 26095785

[B84] Gopalakrishnan V. Spencer C. N. Nezi L. Reuben A. Andrews M. C. Karpinets T. V.. (2018). Gut microbiome modulates response to anti-PD-1 immunotherapy in melanoma patients. Science 359 (6371), 97–103. doi: 10.1126/science.aan4236 29097493 PMC5827966

[B85] Goudarzi M. Mak T. D. Jacobs J. P. Moon B. H. Strawn S. J. Braun J.. (2016). An integrated multi-omic approach to assess radiation injury on the host-microbiome axis. Radiat. Res. 186 (3), 219–234. doi: 10.1667/RR14306.1 27512828 PMC5304359

[B86] Grapin M. Richard C. Limagne E. Boidot R. Morgand V. Bertaut A.. (2019). Optimized fractionated radiotherapy with anti-PD-L1 and anti-TIGIT: a promising new combination. J. Immunother. Cancer. 7 (1), 160. doi: 10.1186/s40425-019-0634-9 31238970 PMC6593525

[B87] Grimaldi A. M. Simeone E. Giannarelli D. Muto P. Falivene S. Borzillo V.. (2014). Abscopal effects of radiotherapy on advanced melanoma patients who progressed after ipilimumab immunotherapy. Oncoimmunology. 3, e28780. doi: 10.4161/onci.28780 25083318 PMC4106166

[B88] Grootaert C. Van de Wiele T. Van Roosbroeck I. Possemiers S. Vercoutter-Edouart A. S. Verstraete W.. (2011). Bacterial monocultures, propionate, butyrate and H2O2 modulate the expression, secretion and structure of the fasting-induced adipose factor in gut epithelial cell lines. Environ. Microbiol. 13 (7), 1778–1789. doi: 10.1111/j.1462-2920.2011.02482.x 21518214

[B89] Gulati M. Singh S. K. Corrie L. Kaur I. P. Chandwani L. (2020). Delivery routes for faecal microbiota transplants: Available, anticipated and aspired. Pharmacol. Res. 159, 104954. doi: 10.1016/j.phrs.2020.104954 32492490

[B90] Ha Y. H. Park D. G. (2010). Effects of DCA on cell cycle proteins in colonocytes. J. Korean Soc. Coloproctol. 26 (4), 254–259. doi: 10.3393/jksc.2010.26.4.254 21152226 PMC2998009

[B91] Habets T. H. Oth T. Houben A. W. Huijskens M. J. Senden-Gijsbers B. L. Schnijderberg M. C.. (2016). Fractionated Radiotherapy with 3 x 8 Gy Induces Systemic Anti-Tumour Responses and Abscopal Tumour Inhibition without Modulating the Humoral Anti-Tumour Response. PloS One 11 (7), e0159515. doi: 10.1371/journal.pone.0159515 27427766 PMC4948777

[B92] Häfner M. F. Debus J. (2016). Radiotherapy for colorectal cancer: current standards and future perspectives. Visc Med. 32 (3), 172–177. doi: 10.1159/000446486 27493944 PMC4945782

[B93] Hale V. L. Jeraldo P. Chen J. Mundy M. Yao J. Priya S.. (2018). Distinct microbes, metabolites, and ecologies define the microbiome in deficient and proficient mismatch repair colorectal cancers. Genome Med. 10 (1), 78. doi: 10.1186/s13073-018-0586-6 30376889 PMC6208080

[B94] Hao Y. Yasmin-Karim S. Moreau M. Sinha N. Sajo E. Ngwa W. (2016). Enhancing radiotherapy for lung cancer using immunoadjuvants delivered in situ from new design radiotherapy biomaterials: a preclinical study. Phys. Med. Biol. 61 (24), N697–n707. doi: 10.1088/1361-6560/61/24/N697 27910826 PMC5209794

[B95] Hauer-Jensen M. Denham J. W. Andreyev H. J. (2014). Radiation enteropathy–pathogenesis, treatment and prevention. Nat. Rev. Gastroenterol. Hepatol. 11 (8), 470–479. doi: 10.1038/nrgastro.2014.46 24686268 PMC4346191

[B96] Hayashi A. Mikami Y. Miyamoto K. Kamada N. Sato T. Mizuno S.. (2017). Intestinal Dysbiosis and Biotin Deprivation Induce Alopecia through Overgrowth of Lactobacillus murinus in Mice. Cell Rep. 20 (7), 1513–1524. doi: 10.1016/j.celrep.2017.07.057 28813664

[B97] Hayes C. S. Shicora A. C. Keough M. P. Snook A. E. Burns M. R. Gilmour S. K. (2014). Polyamine-blocking therapy reverses immunosuppression in the tumor microenvironment. Cancer Immunol. Res. 2 (3), 274–285. doi: 10.1158/2326-6066.CIR-13-0120-T 24778323 PMC4101915

[B98] He Y. Fu L. Li Y. Wang W. Gong M. Zhang J.. (2021). Gut microbial metabolites facilitate anticancer therapy efficacy by modulating cytotoxic CD8(+) T cell immunity. Cell Metab. 33 (5), 988–1000.e7. doi: 10.1016/j.cmet.2021.03.002 33761313

[B99] Hill C. Guarner F. Reid G. Gibson G. R. Merenstein D. J. Pot B.. (2014). Expert consensus document. The International Scientific Association for Probiotics and Prebiotics consensus statement on the scope and appropriate use of the term probiotic. Nat. Rev. Gastroenterol. Hepatol. 11 (8), 506–514. doi: 10.1038/nrgastro.2014.66 24912386

[B100] Hou X. Zheng Z. Wei J. Zhao L. (2022). Effects of gut microbiota on immune responses and immunotherapy in colorectal cancer. Front. Immunol. 13, 1030745. doi: 10.3389/fimmu.2022.1030745 36426359 PMC9681148

[B101] Huang J. Zheng X. Kang W. Hao H. Mao Y. Zhang H.. (2022). Metagenomic and metabolomic analyses reveal synergistic effects of fecal microbiota transplantation and anti-PD-1 therapy on treating colorectal cancer. Front. Immunol. 13, 874922. doi: 10.3389/fimmu.2022.874922 35911731 PMC9336524

[B102] Hullar M. A. Burnett-Hartman A. N. Lampe J. W. (2014). Gut microbes, diet, and cancer. Cancer Treat Res. 159, 377–399. doi: 10.1007/978-3-642-38007-5_22 24114492 PMC4121395

[B103] Jain T. Sharma P. Are A. C. Vickers S. M. Dudeja V. (2021). New insights into the cancer-microbiome-immune axis: decrypting a decade of discoveries. Front. Immunol. 12, 622064. doi: 10.3389/fimmu.2021.622064 33708214 PMC7940198

[B104] Jandhyala S. M. Talukdar R. Subramanyam C. Vuyyuru H. Sasikala M. Nageshwar Reddy D. (2015). Role of the normal gut microbiota. World J. Gastroenterol. 21 (29), 8787–8803. doi: 10.3748/wjg.v21.i29.8787 26269668 PMC4528021

[B105] Javed M. Althwanay A. Ahsan F. Oliveri F. Goud H. K. Mehkari Z.. (2020). Role of vitamin D in colorectal cancer: A holistic approach and review of the clinical utility. Cureus 12 (9), e10734. doi: 10.7759/cureus.10734 33145139 PMC7599058

[B106] Jeggo P. A. Pearl L. H. Carr A. M. (2016). DNA repair, genome stability and cancer: a historical perspective. Nat. Rev. Cancer. 16 (1), 35–42. doi: 10.1038/nrc.2015.4 26667849

[B107] Jess T. Rungoe C. Peyrin-Biroulet L. (2012). Risk of colorectal cancer in patients with ulcerative colitis: a meta-analysis of population-based cohort studies. Clin. Gastroenterol. Hepatol. 10 (6), 639–645. doi: 10.1016/j.cgh.2012.01.010 22289873

[B108] Jiang Y. Ben Q. Shen H. Lu W. Zhang Y. Zhu J. (2011). Diabetes mellitus and incidence and mortality of colorectal cancer: a systematic review and meta-analysis of cohort studies. Eur. J. Epidemiol. 26 (11), 863–876. doi: 10.1007/s10654-011-9617-y 21938478

[B109] Jin M. Wu J. Shi L. Zhou B. Shang F. Chang X.. (2022). Gut microbiota distinct between colorectal cancers with deficient and proficient mismatch repair: A study of 230 CRC patients. Front. Microbiol. 13, 993285. doi: 10.3389/fmicb.2022.993285 36312959 PMC9607965

[B110] Johnson C. H. Dejea C. M. Edler D. Hoang L. T. Santidrian A. F. Felding B. H.. (2015). Metabolism links bacterial biofilms and colon carcinogenesis. Cell Metab. 21 (6), 891–897. doi: 10.1016/j.cmet.2015.04.011 25959674 PMC4456201

[B111] Kalasabail S. Engelman J. Zhang L. Y. El-Omar E. (2021). Yim HCH. A perspective on the role of microbiome for colorectal cancer treatment. Cancers (Basel) 13 (18):4623. doi: 10.3390/cancers13184623 34572850 PMC8468110

[B112] Kalbasi A. Komar C. Tooker G. M. Liu M. Lee J. W. Gladney W. L.. (2017). Tumor-derived CCL2 mediates resistance to radiotherapy in pancreatic ductal adenocarcinoma. Clin. Cancer Res. 23 (1), 137–148. doi: 10.1158/1078-0432.CCR-16-0870 27354473 PMC5195913

[B113] Kang X. Zhang R. Kwong T. N. Lui R. N. Wu W. K. Sung J. J.. (2021). Serrated neoplasia in the colorectum: gut microbiota and molecular pathways. Gut Microbes 13 (1), 1–12. doi: 10.1080/19490976.2020.1863135 PMC7781617 33382354

[B114] Kaźmierczak-Siedlecka K. Daca A. Fic M. van de Wetering T. Folwarski M. Makarewicz W. (2020). Therapeutic methods of gut microbiota modification in colorectal cancer management - fecal microbiota transplantation, prebiotics, probiotics, and synbiotics. Gut Microbes 11 (6), 1518–1530. doi: 10.1080/19490976.2020.1764309 32453670 PMC7524363

[B115] Keam S. Gill S. Ebert M. A. Nowak A. K. Cook A. M. (2020). Enhancing the efficacy of immunotherapy using radiotherapy. Clin. Transl. Immunol. 9 (9), e1169. doi: 10.1002/cti2.1169 PMC7507442 32994997

[B116] Kelly C. R. Ihunnah C. Fischer M. Khoruts A. Surawicz C. Afzali A.. (2014). Fecal microbiota transplant for treatment of *Clostridium* difficile infection in immunocompromised patients. Am. J. Gastroenterol. 109 (7), 1065–1071. doi: 10.1038/ajg.2014.133 24890442 PMC5537742

[B117] Keramidaris D. Koronakis N. Lagoudianakis E. E. Pappas A. Koukoutsis I. Chrysikos I.. (2013). Procalcitonin in patients with colorectal cancer. J. buon. 18 (3), 623–628. 24065474

[B118] Kim S. K. Cho S. W. (2022). The evasion mechanisms of cancer immunity and drug intervention in the tumor microenvironment. Front. Pharmacol. 13, 868695. doi: 10.3389/fphar.2022.868695 35685630 PMC9171538

[B119] Kim Y. S. Kim J. Park S. J. (2015). High-throughput 16S rRNA gene sequencing reveals alterations of mouse intestinal microbiota after radiotherapy. Anaerobe. 33, 1–7. doi: 10.1016/j.anaerobe.2015.01.004 25600706

[B120] Kim J. K. Wu C. Del Latto M. Gao Y. Choi S. H. Kierstead M.. (2022). An immunocompetent rectal cancer model to study radiation therapy. Cell Rep. Methods 2 (12), 100353. doi: 10.1016/j.crmeth.2022.100353 36590695 PMC9795330

[B121] Klug F. Prakash H. Huber P. E. Seibel T. Bender N. Halama N.. (2013). Low-dose irradiation programs macrophage differentiation to an iNO⁺/M1 phenotype that orchestrates effective T cell immunotherapy. Cancer Cell 24 (5), 589–602. doi: 10.1016/j.ccr.2013.09.014 24209604

[B122] Koller K. M. Mackley H. B. Liu J. Wagner H. Talamo G. Schell T. D.. (2017). Improved survival and complete response rates in patients with advanced melanoma treated with concurrent ipilimumab and radiotherapy versus ipilimumab alone. Cancer Biol. Ther. 18 (1), 36–42. doi: 10.1080/15384047.2016.1264543 27905824 PMC5323007

[B123] Kroemer G. Galluzzi L. Kepp O. Zitvogel L. (2013). Immunogenic cell death in cancer therapy. Annu. Rev. Immunol. 31 (1), 51–72. doi: 10.1146/annurev-immunol-032712-100008 23157435

[B124] Kuipers E. J. Grady W. M. Lieberman D. Seufferlein T. Sung J. J. Boelens P. G.. (2015). Colorectal cancer. Nat. Rev. Dis. Primers. 1, 15065. doi: 10.1038/nrdp.2015.65 27189416 PMC4874655

[B125] Kumar A. Gautam V. Sandhu A. Rawat K. Sharma A. Saha L. (2023). Current and emerging therapeutic approaches for colorectal cancer: A comprehensive review. World J. Gastrointest Surg. 15 (4), 495–519. doi: 10.4240/wjgs.v15.i4.495 37206081 PMC10190721

[B126] Kuwahara Y. Oikawa T. Ochiai Y. Roudkenar M. H. Fukumoto M. Shimura T.. (2011). Enhancement of autophagy is a potential modality for tumors refractory to radiotherapy. Cell Death Dis. 2 (6), e177. doi: 10.1038/cddis.2011.56 21716292 PMC3168998

[B127] Le D. T. Durham J. N. Smith K. N. Wang H. Bartlett B. R. Aulakh L. K.. (2017). Mismatch repair deficiency predicts response of solid tumors to PD-1 blockade. Science. 357 (6349), 409–413. doi: 10.1126/science.aan6733 28596308 PMC5576142

[B128] Leeming E. R. Johnson A. J. Spector T. D. Le Roy C. I. (2019). Effect of diet on the gut microbiota: rethinking intervention duration. Nutrients 11 (12), 2862. doi: 10.3390/nu11122862 31766592 PMC6950569

[B129] Lenz H. J. Van Cutsem E. Luisa Limon M. Wong K. Y. M. Hendlisz A. Aglietta M.. (2022). First-line nivolumab plus low-dose ipilimumab for microsatellite instability-high/mismatch repair-deficient metastatic colorectal cancer: the phase II checkMate 142 study. J. Clin. Oncol. 40 (2), 161–170. doi: 10.1200/JCO.21.01015 34637336

[B130] Li N. Chen H. Cheng Y. Xu F. Ruan G. Ying S.. (2021). Fecal microbiota transplantation relieves gastrointestinal and autism symptoms by improving the gut microbiota in an open-label study. Front. Cell Infect. Microbiol. 11, 759435. doi: 10.3389/fcimb.2021.759435 34737978 PMC8560686

[B131] Li Y. Dong J. Xiao H. Zhang S. Wang B. Cui M.. (2020). Gut commensal derived-valeric acid protects against radiation injuries. Gut Microbes 11 (4), 789–806. doi: 10.1080/19490976.2019.1709387 31931652 PMC7524389

[B132] Li Q. Hu W. Liu W. X. Zhao L. Y. Huang D. Liu X. D.. (2021). Streptococcus thermophilus inhibits colorectal tumorigenesis through secreting β-galactosidase. Gastroenterology 160 (4), 1179–93.e14. doi: 10.1053/j.gastro.2020.09.003 32920015

[B133] Li Y. Tinoco R. Elmén L. Segota I. Xian Y. Fujita Y.. (2019). Gut microbiota dependent anti-tumor immunity restricts melanoma growth in Rnf5(-/-) mice. Nat. Commun. 10 (1), 1492. doi: 10.1038/s41467-019-09525-y 30940817 PMC6445090

[B134] Lin K. J. Cheung W. Y. Lai J. Y. Giovannucci E. L. (2012). The effect of estrogen vs. combined estrogen-progestogen therapy on the risk of colorectal cancer. Int. J. Cancer. 130 (2), 419–430. doi: 10.1002/ijc.26026 21365647

[B135] Liu Y. Lau H. C. Cheng W. Y. Yu J. (2023). Gut microbiome in colorectal cancer: clinical diagnosis and treatment. Genomics Proteomics Bioinf. 21 (1), 84–96. doi: 10.1016/j.gpb.2022.07.002 PMC10372906 35914737

[B136] Liu J. Liu C. Yue J. (2021). Radiotherapy and the gut microbiome: facts and fiction. Radiat. Oncol. 16 (1), 9. doi: 10.1186/s13014-020-01735-9 33436010 PMC7805150

[B137] Liu T. Su D. Lei C. Liu Z. (2022). Treatment of radiation enteritis with fecal transplantation. Am. Surg. 31348221091954, 1863135. doi: 10.1177/00031348221091954 35695221

[B138] Liu X. Zhou Y. Wang S. Guan H. Hu S. Huang R.. (2019). Impact of low-dose ionising radiation on the composition of the gut microbiota of mice. Toxicol. Sci. 171 (1), 258–268. doi: 10.1093/toxsci/kfz144 31236581

[B139] Loddo I. Romano C. (2015). Inflammatory bowel disease: genetics, epigenetics, and pathogenesis. Front. Immunol. 6, 551. doi: 10.3389/fimmu.2015.00551 26579126 PMC4629465

[B140] Long X. Wong C. C. Tong L. Chu E. S. H. Ho Szeto C. Go M. Y. Y.. (2019). Peptostreptococcus anaerobius promotes colorectal carcinogenesis and modulates tumour immunity. Nat. Microbiol. 4 (12), 2319–2330. doi: 10.1038/s41564-019-0541-3 31501538

[B141] Lozupone C. A. Stombaugh J. I. Gordon J. I. Jansson J. K. Knight R. (2012). Diversity, stability and resilience of the human gut microbiota. Nature 489 (7415), 220–230. doi: 10.1038/nature11550 22972295 PMC3577372

[B142] Ma Y. Yang Y. Wang F. Zhang P. Shi C. Zou Y.. (2013). Obesity and risk of colorectal cancer: a systematic review of prospective studies. PloS One 8 (1), e53916. doi: 10.1371/journal.pone.0053916 23349764 PMC3547959

[B143] Ma X. Zhou Z. Zhang X. Fan M. Hong Y. Feng Y.. (2020). Sodium butyrate modulates gut microbiota and immune response in colorectal cancer liver metastatic mice. Cell Biol. Toxicol. 36 (5), 509–515. doi: 10.1007/s10565-020-09518-4 32172331

[B144] Maby P. Galon J. Latouche J. B. (2016). Frameshift mutations, neoantigens and tumor-specific CD8(+) T cells in microsatellite unstable colorectal cancers. Oncoimmunology 5 (5), e1115943. doi: 10.1080/2162402X.2015.1115943 27467916 PMC4910722

[B145] Maddocks O. D. K. Athineos D. Cheung E. C. Lee P. Zhang T. van den Broek N. J. F.. (2017). Modulating the therapeutic response of tumours to dietary serine and glycine starvation. Nature 544 (7650), 372–376. doi: 10.1038/nature22056 28425994

[B146] Mager L. F. Burkhard R. Pett N. Cooke N. C. A. Brown K. Ramay H.. (2020). Microbiome-derived inosine modulates response to checkpoint inhibitor immunotherapy. Science 369 (6510), 1481–1489. doi: 10.1126/science.abc3421 32792462

[B147] Maisonneuve C. Irrazabal T. Martin A. Girardin S. E. Philpott D. J. (2018). The impact of the gut microbiome on colorectal cancer. Annu. Rev. Cancer Biol. 2 (1), 229–249. doi: 10.1146/annurev-cancerbio-030617-050240

[B148] Manichanh C. Varela E. Martinez C. Antolin M. Llopis M. Doré J.. (2008). The gut microbiota predispose to the pathophysiology of acute postradiotherapy diarrhea. Am. J. Gastroenterol. 103 (7), 1754–1761. doi: 10.1111/j.1572-0241.2008.01868.x 18564125

[B149] Mármol I. Sánchez-de-Diego C. Pradilla Dieste A. Cerrada E. Rodriguez Yoldi M. J. (2017). Colorectal carcinoma: A general overview and future perspectives in colorectal cancer. Int. J. Mol. Sci. 18 (1), 197. doi: 10.3390/ijms18010197 28106826 PMC5297828

[B150] Martins F. Sofiya L. Sykiotis G. P. Lamine F. Maillard M. Fraga M.. (2019). Adverse effects of immune-checkpoint inhibitors: epidemiology, management and surveillance. Nat. Rev. Clin. Oncol. 16 (9), 563–580. doi: 10.1038/s41571-019-0218-0 31092901

[B151] Matson V. Fessler J. Bao R. Chongsuwat T. Zha Y. Alegre M. L.. (2018). The commensal microbiome is associated with anti-PD-1 efficacy in metastatic melanoma patients. Science 359 (6371), 104–108. doi: 10.1126/science.aao3290 29302014 PMC6707353

[B152] McLaughlin M. Patin E. C. Pedersen M. Wilkins A. Dillon M. T. Melcher A. A.. (2020). Inflammatory microenvironment remodelling by tumour cells after radiotherapy. Nat. Rev. Cancer 20 (4), 203–217. doi: 10.1038/s41568-020-0246-1 32161398

[B153] McQuade R. M. Stojanovska V. Bornstein J. C. Nurgali K. (2017). Colorectal cancer chemotherapy: the evolution of treatment and new approaches. Curr. Med. Chem. 24 (15), 1537–1557. doi: 10.2174/0929867324666170111152436 28079003

[B154] Mete M. Ünsal Ü. Aydemir I. Sönmez P. K. Tuglu M. I. (2019). Punicic acid inhibits glioblastoma migration and proliferation via the PI3K/AKT1/mTOR signaling pathway. Anticancer Agents Med. Chem. 19 (9), 1120–1131. doi: 10.2174/1871520619666190405112507 30950355

[B155] Mima K. Nishihara R. Qian Z. R. Cao Y. Sukawa Y. Nowak J. A.. (2016). Fusobacterium nucleatum in colorectal carcinoma tissue and patient prognosis. Gut. 65 (12), 1973–1980. doi: 10.1136/gutjnl-2015-310101 26311717 PMC4769120

[B156] Mole R. H. (1953). Whole body irradiation; radiobiology or medicine? Br. J. Radiol. 26 (305), 234–241. doi: 10.1259/0007-1285-26-305-234 13042090

[B157] Mondini M. Levy A. Meziani L. Milliat F. Deutsch E. (2020). Radiotherapy-immunotherapy combinations - perspectives and challenges. Mol. Oncol. 14 (7), 1529–1537. doi: 10.1002/1878-0261.12658 32112478 PMC7332212

[B158] Monjazeb A. M. Giobbie-Hurder A. Lako A. Thrash E. M. Brennick R. C. Kao K. Z.. (2021). A randomized trial of combined PD-L1 and CTLA-4 inhibition with targeted low-dose or hypofractionated radiation for patients with metastatic colorectal cancer. Clin. Cancer Res. 27 (9), 2470–2480. doi: 10.1158/1078-0432.CCR-20-4632 33568343 PMC8102320

[B159] Muroyama Y. Nirschl T. R. Kochel C. M. Lopez-Bujanda Z. Theodros D. Mao W.. (2017). Stereotactic radiotherapy increases functionally suppressive regulatory T cells in the tumor microenvironment. Cancer Immunol. Res. 5 (11), 992–1004. doi: 10.1158/2326-6066.CIR-17-0040 28970196 PMC5793220

[B160] Muthusamy T. Cordes T. Handzlik M. K. You L. Lim E. W. Gengatharan J.. (2020). Serine restriction alters sphingolipid diversity to constrain tumour growth. Nature. 586 (7831), 790–795. doi: 10.1038/s41586-020-2609-x 32788725 PMC7606299

[B161] Nagao-Kitamoto H. Kitamoto S. Kamada N. (2022). Inflammatory bowel disease and carcinogenesis. Cancer Metastasis Rev. 41 (2), 301–316. doi: 10.1007/s10555-022-10028-4 35416564

[B162] Nam Y. D. Kim H. J. Seo J. G. Kang S. W. Bae J. W. (2013). Impact of pelvic radiotherapy on gut microbiota of gynecological cancer patients revealed by massive pyrosequencing. PloS One 8 (12), e82659. doi: 10.1371/journal.pone.0082659 24367534 PMC3867375

[B163] Neff C. P. Rhodes M. E. Arnolds K. L. Collins C. B. Donnelly J. Nusbacher N.. (2016). Diverse intestinal bacteria contain putative zwitterionic capsular polysaccharides with anti-inflammatory properties. Cell Host Microbe 20 (4), 535–547. doi: 10.1016/j.chom.2016.09.002 27693306 PMC5113727

[B164] Ngwa W. Irabor O. C. Schoenfeld J. D. Hesser J. Demaria S. Formenti S. C. (2018). Using immunotherapy to boost the abscopal effect. Nat. Rev. Cancer. 18 (5), 313–322. doi: 10.1038/nrc.2018.6 29449659 PMC5912991

[B165] Nicolas A. M. Pesic M. Engel E. Ziegler P. K. Diefenhardt M. Kennel K. B.. (2022a). Inflammatory fibroblasts mediate resistance to neoadjuvant therapy in rectal cancer. Cancer Cell. 40 (2), 168–84.e13. doi: 10.1016/j.ccell.2022.01.004 35120600

[B166] Nicolas A. M. Pesic M. Rödel F. Fokas E. Greten F. R. (2022b). Image-guided radiotherapy in an orthotopic mouse model of rectal cancer. STAR Protoc. 3 (4), 101749. doi: 10.1016/j.xpro.2022.101749 36206161 PMC9547293

[B167] Nishida A. Inoue R. Inatomi O. Bamba S. Naito Y. Andoh A. (2018). Gut microbiota in the pathogenesis of inflammatory bowel disease. Clin. J. Gastroenterol. 11 (1), 1–10. doi: 10.1007/s12328-017-0813-5 29285689

[B168] Novita Sari I. Setiawan T. Seock Kim K. Toni Wijaya Y. Won Cho K. Young Kwon H. (2021). Metabolism and function of polyamines in cancer progression. Cancer Lett. 519, 91–104. doi: 10.1016/j.canlet.2021.06.020 34186159

[B169] Oh S. F. Praveena T. Song H. Yoo J. S. Jung D. J. Erturk-Hasdemir D.. (2021). Host immunomodulatory lipids created by symbionts from dietary amino acids. Nature 600 (7888), 302–307. doi: 10.1038/s41586-021-04083-0 34759313 PMC8999822

[B170] Olson B. Li Y. Lin Y. Liu E. T. Patnaik A. (2018). Mouse models for cancer immunotherapy research. Cancer Discov. 8 (11), 1358–1365. doi: 10.1158/2159-8290.CD-18-0044 30309862 PMC8725605

[B171] Overman M. J. McDermott R. Leach J. L. Lonardi S. Lenz H. J. Morse M. A.. (2017). Nivolumab in patients with metastatic DNA mismatch repair-deficient or microsatellite instability-high colorectal cancer (CheckMate 142): an open-label, multicentre, phase 2 study. Lancet Oncol. 18 (9), 1182–1191. doi: 10.1016/S1470-2045(17)30422-9 28734759 PMC6207072

[B172] Panda A. Mehnert J. M. Hirshfield K. M. Riedlinger G. Damare S. Saunders T.. (2018). Immune activation and benefit from avelumab in EBV-positive gastric cancer. J. Natl. Cancer Inst. 110 (3), 316–320. doi: 10.1093/jnci/djx213 29155997 PMC6658862

[B173] Paramsothy S. Kamm M. A. Kaakoush N. O. Walsh A. J. van den Bogaerde J. Samuel D.. (2017). Multidonor intensive faecal microbiota transplantation for active ulcerative colitis: a randomised placebo-controlled trial. Lancet 389 (10075), 1218–1228. doi: 10.1016/S0140-6736(17)30182-4 28214091

[B174] Parikh A. R. Szabolcs A. Allen J. N. Clark J. W. Wo J. Y. Raabe M.. (2021). Radiation therapy enhances immunotherapy response in microsatellite stable colorectal and pancreatic adenocarcinoma in a phase II trial. Nat. Cancer 2 (11), 1124–1135. doi: 10.1038/s43018-021-00269-7 35122060 PMC8809884

[B175] Park J. Goergen C. J. HogenEsch H. Kim C. H. (2016). Chronically elevated levels of short-chain fatty acids induce T cell-mediated ureteritis and hydronephrosis. J. Immunol. 196 (5), 2388–2400. doi: 10.4049/jimmunol.1502046 26819206 PMC4761537

[B176] Park R. Umar S. Kasi A. (2020). Immunotherapy in colorectal cancer: potential of fecal transplant and microbiota-augmented clinical trials. Curr. Colorectal Cancer Rep. 16 (4), 81–88. doi: 10.1007/s11888-020-00456-1 32607098 PMC7325521

[B177] Peng Z. Cheng S. Kou Y. Wang Z. Jin R. Hu H.. (2020). The gut microbiome is associated with clinical response to anti-PD-1/PD-L1 immunotherapy in gastrointestinal cancer. Cancer Immunol. Res. 8 (10), 1251–1261. doi: 10.1158/2326-6066.CIR-19-1014 32855157

[B178] Peng Z. Zhang X. Xie T. Cheng S. Han Z. Wang S.. (2023). Efficacy of fecal microbiota transplantation in patients with anti-PD-1–resistant/refractory gastrointestinal cancers. J. Clin. Oncol. 41 (4_suppl), 389. doi: 10.1200/JCO.2023.41.4_suppl.389

[B179] Perregaux D. G. McNiff P. Laliberte R. Conklyn M. Gabel C. A. (2000). ATP acts as an agonist to promote stimulus-induced secretion of IL-1 beta and IL-18 in human blood. J. Immunol. 165 (8), 4615–4623. doi: 10.4049/jimmunol.165.8.4615 11035104

[B180] Peters B. A. Wilson M. Moran U. Pavlick A. Izsak A. Wechter T.. (2019). Relating the gut metagenome and metatranscriptome to immunotherapy responses in melanoma patients. Genome Med. 11 (1), 61. doi: 10.1186/s13073-019-0672-4 31597568 PMC6785875

[B181] Pham V. T. Dold S. Rehman A. Bird J. K. Steinert R. E. (2021). Vitamins, the gut microbiome and gastrointestinal health in humans. Nutr. Res. 95, 35–53. doi: 10.1016/j.nutres.2021.09.001 34798467

[B182] Pourhoseingholi M. A. (2012). Increased burden of colorectal cancer in Asia. World J. Gastrointest Oncol. 4 (4), 68–70. doi: 10.4251/wjgo.v4.i4.68 22532878 PMC3334381

[B183] Prakash H. Klug F. Nadella V. Mazumdar V. Schmitz-Winnenthal H. Umansky L. (2016). Low doses of gamma irradiation potentially modifies immunosuppressive tumor microenvironment by retuning tumor-associated macrophages: lesson from insulinoma. Carcinogenesis. 37 (3), 301–313. doi: 10.1093/carcin/bgw007 26785731

[B184] Ramai D. Zakhia K. Ofosu A. Ofori E. Reddy M. (2019). Fecal microbiota transplantation: donor relation, fresh or frozen, delivery methods, cost-effectiveness. Ann. Gastroenterol. 32 (1), 30–38. doi: 10.20524/aog.2018.0328 30598589 PMC6302197

[B185] Ramos-Casals M. Brahmer J. R. Callahan M. K. Flores-Chávez A. Keegan N. Khamashta M. A.. (2020). Immune-related adverse events of checkpoint inhibitors. Nat. Rev. Dis. Primers. 6 (1), 38. doi: 10.1038/s41572-020-0160-6 32382051 PMC9728094

[B186] Rebersek M. (2021). Gut microbiome and its role in colorectal cancer. BMC Cancer. 21 (1), 1325. doi: 10.1186/s12885-021-09054-2 34895176 PMC8666072

[B187] Reis Ferreira M. Andreyev H. J. N. Mohammed K. Truelove L. Gowan S. M. Li J.. (2019). Microbiota- and radiotherapy-induced gastrointestinal side-effects (MARS) study: A large pilot study of the microbiome in acute and late-radiation enteropathy. Clin. Cancer Res. 25 (21), 6487–6500. doi: 10.1158/1078-0432.CCR-19-0960 31345839

[B188] Rodríguez-Ruiz M. E. Perez-Gracia J. L. Rodríguez I. Alfaro C. Oñate C. Pérez G.. (2018). Combined immunotherapy encompassing intratumoral poly-ICLC, dendritic-cell vaccination and radiotherapy in advanced cancer patients. Ann. Oncol. 29 (5), 1312–1319. doi: 10.1093/annonc/mdy089 29554212

[B189] Rodriguez-Ruiz M. E. Rodriguez I. Barbes B. Mayorga L. Sanchez-Paulete A. R. Ponz-Sarvise M.. (2017). Brachytherapy attains abscopal effects when combined with immunostimulatory monoclonal antibodies. Brachytherapy 16 (6), 1246–1251. doi: 10.1016/j.brachy.2017.06.012 28838649

[B190] Roger A. Finet A. Boru B. Beauchet A. Mazeron J. J. Otzmeguine Y.. (2018). Efficacy of combined hypo-fractionated radiotherapy and anti-PD-1 monotherapy in difficult-to-treat advanced melanoma patients. Oncoimmunology. 7 (7), e1442166. doi: 10.1080/2162402X.2018.1442166 30034949 PMC6053300

[B191] Routy B. Le Chatelier E. Derosa L. Duong C. P. M. Alou M. T. Daillere R.. (2018). Gut microbiome influences efficacy of PD-1-based immunotherapy against epithelial tumors. Science 359 (6371), 91–97. doi: 10.1038/s41571-018-0006-2 29097494

[B192] Ryu T. Y. Kim K. Han T. S. Lee M. O. Lee J. Choi J.. (2022). Human gut-microbiome-derived propionate coordinates proteasomal degradation via HECTD2 upregulation to target EHMT2 in colorectal cancer. Isme J. 16 (5), 1205–1221. doi: 10.1038/s41396-021-01119-1 34972816 PMC9038766

[B193] Sahly N. Moustafa A. Zaghloul M. Salem T. Z. (2019). Effect of radiotherapy on the gut microbiome in pediatric cancer patients: a pilot study. PeerJ. 7, e7683. doi: 10.7717/peerj.7683 31579590 PMC6761921

[B194] Salminen S. Collado M. C. Endo A. Hill C. Lebeer S. Quigley E. M. M.. (2021). The International Scientific Association of Probiotics and Prebiotics (ISAPP) consensus statement on the definition and scope of postbiotics. Nat. Rev. Gastroenterol. Hepatol. 18 (9), 649–667. doi: 10.1038/s41575-021-00440-6 33948025 PMC8387231

[B195] Sánchez-Alcoholado L. Ramos-Molina B. Otero A. Laborda-Illanes A. Ordóñez R. Medina J. A.. (2020). The role of the gut microbiome in colorectal cancer development and therapy response. Cancers (Basel) 12 (6), 1406. doi: 10.3390/cancers12061406 32486066 PMC7352899

[B196] Sanderson S. M. Gao X. Dai Z. Locasale J. W. (2019). Methionine metabolism in health and cancer: a nexus of diet and precision medicine. Nat. Rev. Cancer. 19 (11), 625–637. doi: 10.1038/s41568-019-0187-8 31515518

[B197] Sarkar A. Lehto S. M. Harty S. Dinan T. G. Cryan J. F. Burnet P. W. J. (2016). Psychobiotics and the manipulation of bacteria-gut-brain signals. Trends Neurosci. 39 (11), 763–781. doi: 10.1016/j.tins.2016.09.002 27793434 PMC5102282

[B198] Segal N. H. Cercek A. Ku G. Wu A. J. Rimner A. Khalil D. N.. (2021). Phase II single-arm study of durvalumab and tremelimumab with concurrent radiotherapy in patients with mismatch repair-proficient metastatic colorectal cancer. Clin. Cancer Res. 27 (8), 2200–2208. doi: 10.1158/1078-0432.CCR-20-2474 33504552 PMC8499477

[B199] Segers C. Mysara M. Claesen J. Baatout S. Leys N. Lebeer S.. (2021). Intestinal mucositis precedes dysbiosis in a mouse model for pelvic irradiation. ISME Commun. 1 (1), 24. doi: 10.1038/s43705-021-00024-0 36737646 PMC9723693

[B200] Segers C. Verslegers M. Baatout S. Leys N. Lebeer S. Mastroleo F. (2019). Food supplements to mitigate detrimental effects of pelvic radiotherapy. Microorganisms 7 (4), 97. doi: 10.3390/microorganisms7040097 30987157 PMC6518429

[B201] Seifert L. Werba G. Tiwari S. Giao Ly N. N. Nguy S. Alothman S.. (2016). Radiation therapy induces macrophages to suppress T-cell responses against pancreatic tumors in mice. Gastroenterology 150 (7), 1659–72.e5. doi: 10.1053/j.gastro.2016.02.070 26946344 PMC4909514

[B202] Shin J. H. Zhang L. Murillo-Sauca O. Kim J. Kohrt H. E. Bui J. D.. (2013). Modulation of natural killer cell antitumor activity by the aryl hydrocarbon receptor. Proc. Natl. Acad. Sci. U. S. A. 110 (30), 12391–12396. doi: 10.1073/pnas.1302856110 23836658 PMC3725066

[B203] Singh R. K. Chang H. W. Yan D. Lee K. M. Ucmak D. Wong K.. (2017). Influence of diet on the gut microbiome and implications for human health. J. Transl. Med. 15 (1), 73. doi: 10.1186/s12967-017-1175-y 28388917 PMC5385025

[B204] Sinicrope F. A. Yang Z. J. (2011). Prognostic and predictive impact of DNA mismatch repair in the management of colorectal cancer. Future Oncol. 7 (3), 467–474. doi: 10.2217/fon.11.5 21417908 PMC3770934

[B205] Sipe L. M. Chaib M. Pingili A. K. Pierre J. F. Makowski L. (2020). Microbiome, bile acids, and obesity: How microbially modified metabolites shape anti-tumor immunity. Immunol. Rev. 295 (1), 220–239. doi: 10.1111/imr.12856 32320071 PMC7841960

[B206] Sobhani I. Amiot A. Le Baleur Y. Levy M. Auriault M. L. Van Nhieu J. T.. (2013). Microbial dysbiosis and colon carcinogenesis: could colon cancer be considered a bacteria-related disease? Therap Adv. Gastroenterol. 6 (3), 215–229. doi: 10.1177/1756283X12473674 PMC3625019 23634186

[B207] Sorolla M. A. Hidalgo I. Sorolla A. Montal R. Pallisé O. Salud A.. (2021). Microenvironmental reactive oxygen species in colorectal cancer: involved processes and therapeutic opportunities. Cancers (Basel) 13 (20), 5037. doi: 10.3390/cancers13205037 34680186 PMC8534037

[B208] Sprooten J. Agostinis P. Garg A. D. (2019). Type I interferons and dendritic cells in cancer immunotherapy. Int. Rev. Cell Mol. Biol. 348, 217–262. doi: 10.1016/bs.ircmb.2019.06.001 31810554

[B209] Sun C. H. Li B. B. Wang B. Zhao J. Zhang X. Y. Li T. T.. (2019). The role of Fusobacterium nucleatum in colorectal cancer: from carcinogenesis to clinical management. Chronic Dis. Transl. Med. 5 (3), 178–187. doi: 10.1016/j.cdtm.2019.09.001 31891129 PMC6926109

[B210] Sun M. F. Shen Y. Q. (2018). Dysbiosis of gut microbiota and microbial metabolites in Parkinson’s Disease. Ageing Res. Rev. 45, 53–61. doi: 10.1016/j.arr.2018.04.004 29705121

[B211] Sung H. Ferlay J. Siegel R. L. Laversanne M. Soerjomataram I. Jemal A.. (2021). Global cancer statistics 2020: GLOBOCAN estimates of incidence and mortality worldwide for 36 cancers in 185 countries. CA Cancer J. Clin. 71 (3), 209–249. doi: 10.3322/caac.21660 33538338

[B212] Tahara T. Yamamoto E. Suzuki H. Maruyama R. Chung W. Garriga J.. (2014). Fusobacterium in colonic flora and molecular features of colorectal carcinoma. Cancer Res. 74 (5), 1311–1318. doi: 10.1158/0008-5472.CAN-13-1865 24385213 PMC4396185

[B213] Tanoue T. Morita S. Plichta D. R. Skelly A. N. Suda W. Sugiura Y.. (2019). A defined commensal consortium elicits CD8 T cells and anti-cancer immunity. Nature 565 (7741), 600–605. doi: 10.1038/s41586-019-0878-z 30675064

[B214] Taylor D. P. Burt R. W. Williams M. S. Haug P. J. Cannon-Albright L. A. (2010). Population-based family history-specific risks for colorectal cancer: a constellation approach. Gastroenterology 138 (3), 877–885. doi: 10.1053/j.gastro.2009.11.044 19932107 PMC2831153

[B215] Theurich S. Rothschild S. I. Hoffmann M. Fabri M. Sommer A. Garcia-Marquez M.. (2016). Local tumor treatment in combination with systemic ipilimumab immunotherapy prolongs overall survival in patients with advanced Malignant melanoma. Cancer Immunol. Res. 4 (9), 744–754. doi: 10.1158/2326-6066.CIR-15-0156 27466265

[B216] Tian H. Ding C. Gong J. Ge X. McFarland L. V. Gu L.. (2016). Treatment of slow transit constipation with fecal microbiota transplantation: A pilot study. J. Clin. Gastroenterol. 50 (10), 865–870. doi: 10.1097/MCG.0000000000000472 26751143

[B217] Toor D. Wsson M. K. Kumar P. Karthikeyan G. Kaushik N. K. Goel C.. (2019). Dysbiosis disrupts gut immune homeostasis and promotes gastric diseases. Int. J. Mol. Sci. 20 (10), 2432. doi: 10.3390/ijms20102432 31100929 PMC6567003

[B218] Twyman-Saint Victor C. Rech A. J. Maity A. Rengan R. Pauken K. E. Stelekati E.. (2015). Radiation and dual checkpoint blockade activate non-redundant immune mechanisms in cancer. Nature 520 (7547), 373–377. doi: 10.1038/nature14292 25754329 PMC4401634

[B219] Vanpouille-Box C. Diamond J. M. Pilones K. A. Zavadil J. Babb J. S. Formenti S. C.. (2015). TGFβ Is a master regulator of radiation therapy-induced antitumor immunity. Cancer Res. 75 (11), 2232–2242. doi: 10.1158/0008-5472.CAN-14-3511 25858148 PMC4522159

[B220] Vanpouille-Box C. Formenti S. C. Demaria S. (2018). Toward precision radiotherapy for use with immune checkpoint blockers. Clin. Cancer Res. 24 (2), 259–265. doi: 10.1158/1078-0432.CCR-16-0037 28751442 PMC5771850

[B221] Veettil S. K. Wong T. Y. Loo Y. S. Playdon M. C. Lai N. M. Giovannucci E. L.. (2021). Role of diet in colorectal cancer incidence: umbrella review of meta-analyses of prospective observational studies. JAMA Netw. Open 4 (2), e2037341. doi: 10.1001/jamanetworkopen.2020.37341 33591366 PMC7887658

[B222] Vucic V. Radovanovic S. Radevic S. Savkovic Z. Mihailovic N. Mihaljevic O.. (2021). Mental health assessment of cancer patients: prevalence and predictive Factors of depression and anxiety. Iran J. Public Health 50 (10), 2017–2027. doi: 10.18502/ijph.v50i10.7502 35223569 PMC8819216

[B223] Walczak K. Turski W. A. Rajtar G. (2014). Kynurenic acid inhibits colon cancer proliferation in *vitro*: effects on signaling pathways. Amino Acids 46 (10), 2393–2401. doi: 10.1007/s00726-014-1790-3 25012123 PMC4168223

[B224] Wallace B. D. Wang H. Lane K. T. Scott J. E. Orans J. Koo J. S.. (2010). Alleviating cancer drug toxicity by inhibiting a bacterial enzyme. Science. 330 (6005), 831–835. doi: 10.1126/science.1191175 21051639 PMC3110694

[B225] Wang Y. J. Fletcher R. Yu J. Zhang L. (2018). Immunogenic effects of chemotherapy-induced tumor cell death. Genes Dis. 5 (3), 194–203. doi: 10.1016/j.gendis.2018.05.003 30320184 PMC6176216

[B226] Wang T. Gnanaprakasam J. N. R. Chen X. Kang S. Xu X. Sun H.. (2020). Inosine is an alternative carbon source for CD8(+)-T-cell function under glucose restriction. Nat. Metab. 2 (7), 635–647. doi: 10.1038/s42255-020-0219-4 32694789 PMC7371628

[B227] Wang J. W. Kuo C. H. Kuo F. C. Wang Y. K. Hsu W. H. Yu F. J.. (2019). Fecal microbiota transplantation: Review and update. J. Formos Med. Assoc. 118 Suppl 1, S23–S31. doi: 10.1016/j.jfma.2018.08.011 30181015

[B228] Wang A. Ling Z. Yang Z. Kiela P. R. Wang T. Wang C.. (2015). Gut microbial dysbiosis may predict diarrhea and fatigue in patients undergoing pelvic cancer radiotherapy: a pilot study. PloS One 10 (5), e0126312. doi: 10.1371/journal.pone.0126312 25955845 PMC4425680

[B229] Wang J. S. Wang H. J. Qian H. L. (2018). Biological effects of radiation on cancer cells. Mil Med. Res. 5 (1), 20. doi: 10.1186/s40779-018-0167-4 29958545 PMC6026344

[B230] Wang Z. Wang Q. Wang X. Zhu L. Chen J. Zhang B.. (2019). Gut microbial dysbiosis is associated with development and progression of radiation enteritis during pelvic radiotherapy. J. Cell Mol. Med. 23 (5), 3747–3756. doi: 10.1111/jcmm.14289 30908851 PMC6484301

[B231] Wang Y. Wiesnoski D. H. Helmink B. A. Gopalakrishnan V. Choi K. DuPont H. L.. (2018). Fecal microbiota transplantation for refractory immune checkpoint inhibitor-associated colitis. Nat. Med. 24 (12), 1804–1808. doi: 10.1038/s41591-018-0238-9 30420754 PMC6322556

[B232] Wang F. Yin Q. Chen L. Davis M. M. (2018). *Bifidobacterium* can mitigate intestinal immunopathology in the context of CTLA-4 blockade. Proc. Natl. Acad. Sci. U. S. A. 115 (1), 157–161. doi: 10.1073/pnas.1712901115 29255057 PMC5776803

[B233] Wassenaar T. M. E. (2018). coli and colorectal cancer: a complex relationship that deserves a critical mindset. Crit. Rev. Microbiol. 44 (5), 619–632. doi: 10.1080/1040841X.2018.1481013 29909724

[B234] Wong S. H. Zhao L. Zhang X. Nakatsu G. Han J. Xu W.. (2017). Gavage of fecal samples from patients with colorectal cancer promotes intestinal carcinogenesis in germ-free and conventional mice. Gastroenterology 153 (6), 1621–33 e6. doi: 10.1053/j.gastro.2017.08.022 28823860

[B235] Xia T. Zhang B. Li Y. Fang B. Zhu X. Xu B.. (2020). New insight into 20(S)-ginsenoside Rh2 against T-cell acute lymphoblastic leukemia associated with the gut microbiota and the immune system. Eur. J. Med. Chem. 203, 112582. doi: 10.1016/j.ejmech.2020.112582 32682197

[B236] Xiao H. W. Cui M. Li Y. Dong J. L. Zhang S. Q. Zhu C. C.. (2020). Gut microbiota-derived indole 3-propionic acid protects against radiation toxicity via retaining acyl-CoA-binding protein. Microbiome 8 (1), 69. doi: 10.1186/s40168-020-00845-6 32434586 PMC7241002

[B237] Xu J. Escamilla J. Mok S. David J. Priceman S. West B.. (2013). CSF1R signaling blockade stanches tumor-infiltrating myeloid cells and improves the efficacy of radiotherapy in prostate cancer. Cancer Res. 73 (9), 2782–2794. doi: 10.1158/0008-5472.CAN-12-3981 23418320 PMC4097014

[B238] Xu X. Lv J. Guo F. Li J. Jia Y. Jiang D.. (2020). Gut microbiome influences the efficacy of PD-1 antibody immunotherapy on MSS-type colorectal Cancer via metabolic pathway. Front. Microbiol. 11, 814. doi: 10.3389/fmicb.2020.00814 32425919 PMC7212380

[B239] Xu D. Tian Y. Xia Q. Ke B. (2021). The cGAS-STING pathway: novel perspectives in liver diseases. Front. Immunol. 12, 682736. doi: 10.3389/fimmu.2021.682736 33995425 PMC8117096

[B240] Xu R. Wang Q. Li L. (2015). A genome-wide systems analysis reveals strong link between colorectal cancer and trimethylamine N-oxide (TMAO), a gut microbial metabolite of dietary meat and fat. BMC Genomics 16 Suppl 7 (Suppl 7), S4. doi: 10.1186/1471-2164-16-S7-S4 PMC4474417 26100814

[B241] Yang C. Mogno I. Contijoch E. J. Borgerding J. N. Aggarwala V. Li Z.. (2020). Fecal igA levels are determined by strain-level differences in bacteroides ovatus and are modifiable by gut microbiota manipulation. Cell Host Microbe 27 (3), 467–75.e6. doi: 10.1016/j.chom.2020.01.016 32075742 PMC7213796

[B242] Yasuda K. Nirei T. Tsuno N. H. Nagawa H. Kitayama J. (2011). Intratumoral injection of interleukin-2 augments the local and abscopal effects of radiotherapy in murine rectal cancer. Cancer Sci. 102 (7), 1257–1263. doi: 10.1111/j.1349-7006.2011.01940.x 21443690

[B243] Young K. H. Baird J. R. Savage T. Cottam B. Friedman D. Bambina S.. (2016). Optimizing timing of immunotherapy improves control of tumors by hypofractionated radiation therapy. PloS One 11 (6), e0157164. doi: 10.1371/journal.pone.0157164 27281029 PMC4900555

[B244] Yu T. Guo F. Yu Y. Sun T. Ma D. Han J.. (2017). Fusobacterium nucleatum promotes chemoresistance to colorectal cancer by modulating autophagy. Cell. 170 (3), 548–63.e16. doi: 10.1016/j.cell.2017.07.008 28753429 PMC5767127

[B245] Yu I. Wu R. Tokumaru Y. Terracina K. P. Takabe K. (2022). The role of the microbiome on the pathogenesis and treatment of colorectal cancer. Cancers (Basel). 14 (22), 5685. doi: 10.3390/cancers14225685 36428777 PMC9688177

[B246] Yuan G. Tan M. Chen X. (2021). Punicic acid ameliorates obesity and liver steatosis by regulating gut microbiota composition in mice. Food Funct. 12 (17), 7897–7908. doi: 10.1039/D1FO01152A 34241611

[B247] Zhang Z. Liu X. Chen D. Yu J. (2022). Radiotherapy combined with immunotherapy: the dawn of cancer treatment. Signal Transduct Target Ther. 7 (1), 258. doi: 10.1038/s41392-022-01102-y 35906199 PMC9338328

[B248] Zhang Y. Yu H. Zhang J. Gao H. Wang S. Li S.. (2021). Cul4A-DDB1-mediated monoubiquitination of phosphoglycerate dehydrogenase promotes colorectal cancer metastasis via increased S-adenosylmethionine. J. Clin. Invest. 131 (21), e146187. doi: 10.1172/JCI146187 34720086 PMC8553555

[B249] Zhao Y. Zhang J. Han X. Fan S. (2019). Total body irradiation induced mouse small intestine senescence as a late effect. J. Radiat. Res. 60 (4), 442–450. doi: 10.1093/jrr/rrz026 31165161 PMC6641339

[B250] Zheng Y. M. He X. X. Xia H. H. Yuan Y. Xie W. R. Cai J. Y.. (2020). Multi-donor multi-course faecal microbiota transplantation relieves the symptoms of chronic hemorrhagic radiation proctitis: A case report. Med. (Baltimore). 99 (39), e22298. doi: 10.1097/MD.0000000000022298 PMC7523865 32991434

[B251] Zheng D. Liwinski T. Elinav E. (2020). Interaction between microbiota and immunity in health and disease. Cell Res. 30 (6), 492–506. doi: 10.1038/s41422-020-0332-7 32433595 PMC7264227

[B252] Zhuo Q. Yu B. Zhou J. Zhang J. Zhang R. Xie J.. (2019). Lysates of Lactobacillus acidophilus combined with CTLA-4-blocking antibodies enhance antitumor immunity in a mouse colon cancer model. Sci. Rep. 9 (1), 20128. doi: 10.1038/s41598-019-56661-y 31882868 PMC6934597

[B253] Zitvogel L. Daillère R. Roberti M. P. Routy B. Kroemer G. (2017). Anticancer effects of the microbiome and its products. Nat. Rev. Microbiol. 15 (8), 465–478. doi: 10.1038/nrmicro.2017.44 28529325

[B254] Zitvogel L. Galluzzi L. Kepp O. Smyth M. J. Kroemer G. (2015). Type I interferons in anticancer immunity. Nat. Rev. Immunol. 15 (7), 405–414. doi: 10.1038/nri3845 26027717

[B255] Zou S. Fang L. Lee M. H. (2018). Dysbiosis of gut microbiota in promoting the development of colorectal cancer. Gastroenterol. Rep. (Oxf). 6 (1), 1–12. doi: 10.1093/gastro/gox031 29479437 PMC5806407

